# Inheritance of Material Wealth in a Natural Population

**DOI:** 10.1111/ele.14505

**Published:** 2024-12-31

**Authors:** Murielle Ålund, S. Eryn McFarlane, Arild Husby, Jonas Knape, Tomas Pärt, Päivi Sirkiä, Franz J. Weissing, David Wheatcroft, Yishu Zhu, Anna Qvarnström

**Affiliations:** ^1^ Division of Animal Ecology, Department of Ecology and Genetics (IEG) Uppsala University Uppsala Sweden; ^2^ Department of Biology York University Toronto Ontario Canada; ^3^ Division of Evolutionary Biology, Department of Ecology and Genetics (IEG) Uppsala University Uppsala Sweden; ^4^ Department of Ecology, Swedish University of Agricultural Sciences SLU Uppsala Sweden; ^5^ Finnish Environment Institute Helsinki Finland; ^6^ Groningen Institute for Evolutionary Life Sciences University of Groningen Groningen The Netherlands; ^7^ Department of Zoology Stockholm University Stockholm Sweden

**Keywords:** animal breeding model, evolutionary rescue, GWAS, heritability, landscape genomics, long‐term study, maladaptation, philopatry, site fidelity, social inheritance

## Abstract

Evolutionary adaptation occurs when individuals vary in access to fitness‐relevant resources and these differences in ‘material wealth’ are heritable. It is typically assumed that the inheritance of material wealth reflects heritable variation in the phenotypic abilities needed to acquire material wealth. We scrutinise this assumption by investigating additional mechanisms underlying the inheritance of material wealth in collared flycatchers. A genome‐wide association analysis reveals a high genomic heritability (*h* ^2^ = 0.405 ± 0.08) of access to caterpillar larvae, a fitness‐relevant resource, in the birds' breeding territories. However, we find little evidence for heritable variation in phenotypic abilities needed to acquire this material wealth. Instead, combined evidence from simulations, experimental and long‐term monitoring data indicate that inheritance of material wealth is largely explained by philopatry causing a within‐population genetic structure across a heterogeneous landscape. Therefore, allelic variants associated with high material wealth may spread in the population without having causal connections to traits promoting local adaptation.

## Graphical abstract

In this study, we use a combination of genomic and phenotypic approaches to test whether material wealth is heritable and to investigate possible underlying mechanisms of inheritance in a monitored population of collared flycatchers (*Ficedula albicollis*). We find surprisingly high genomic and quantitative genetic heritability of material wealth. By using long‐term data and cross‐fostering experiments we can conclude that a major non‐genetic basis to this inheritance of wealth largely that arises as a consequence of a heterogeneous distribution of material wealth across the landscape in combination with strong philopatry.

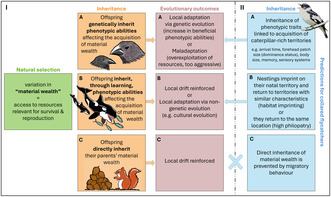

## Introduction

**FIGURE 1:**
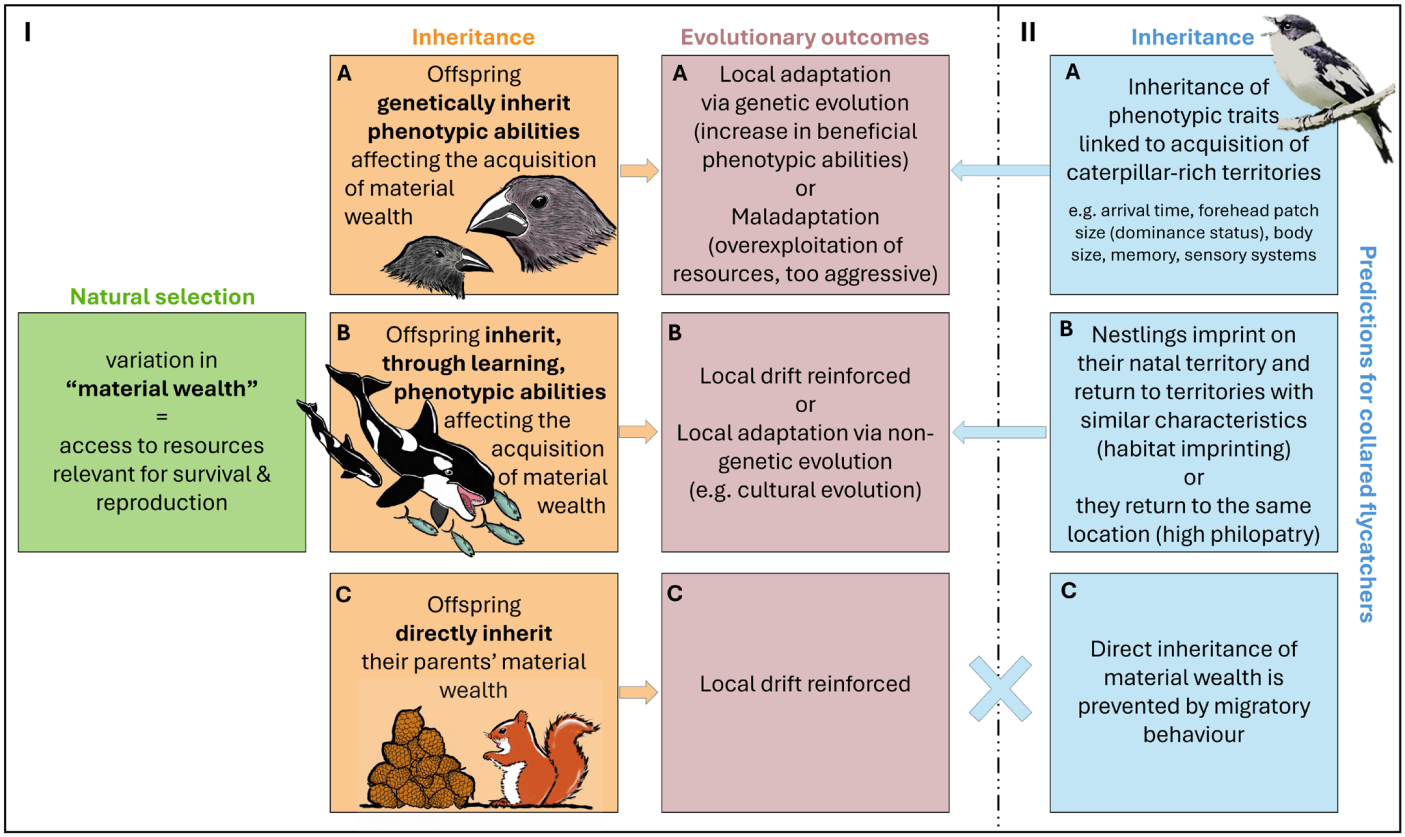
(I) Theoretical expectations. The evolutionary outcome of natural selection caused by variation in material wealth depends on inheritance patterns. Inheritance of material wealth may occur through three main and non‐mutually exclusive pathways: (A) offspring indirectly inherit material wealth through (mostly genetic) inheritance of phenotypic abilities used to acquire wealth. (B) offspring indirectly inherit material wealth through (mostly non‐genetic) inheritance of phenotypic abilities used to acquire wealth, for example, by learning skills from their parents such as hunting prey or by imprinting on local site characteristics, (C) offspring directly inherit material wealth by, for example, being allowed to settle within their parent's territory or by obtaining their parent's social rank. All these ways of inheritance of material wealth can lead to associations between alleles and material wealth. However, inheritance of wealth through mechanisms (B) and (C) can lead to such association regardless of the function of these alleles. Alleles that become associated with high material wealth can increase in frequency in the population because material wealth ensures reproduction even if these alleles lack causal effects on acquisition of material wealth. Therefore, evolutionary outcomes depend on the mechanism of inheritance of wealth. (II) In this study we measure material wealth as access to caterpillar larvae within the breeding territories of collared flycatchers. Direct inheritance of material wealth (C) is not possible in this case because flycatchers are migratory birds that need to establish new breeding territories when they arrive to breed each spring. Indirect inheritance of this material wealth may occur through all phenotypic traits that influence the birds' acquisition of breeding territories with various levels of abundance of caterpillar larvae necessary to produce offspring. These phenotypic material wealth acquisition traits could be: (A) ‘classic’ fitness‐related traits with a fairly high genetic basis such as forehead patch size (dominance badge) and also cognitive traits needed to find and evaluate resource quality that are difficult to measure in natural populations, (B) traits that are mostly non‐genetically inherited through learning such as preferences for the characteristics of the natal site (causing natal philopatry) or preferences for specific habitat characteristics of the natal site that are likely to influence the similarity between natal and adult material wealth.

Variation in access to limited material resources needed for survival and reproduction, such as food, water and shelter (hereafter ‘material wealth’, Box 1) constitutes an important source of natural selection (Darwin 1859). However, individual variation in material wealth is rarely directly measured in natural populations (but see Chase, Douady, and Padilla 2020). Much research is instead directed towards identifying phenotypic traits underlying individual variation in the ability to acquire material wealth (Enbody et al. 2023; Pärt and Qvarnström 1997; Ridley 2003), often referred to as ‘fitness‐related traits’. Great efforts are also spent on estimating the heritability of such traits to, for example, predict whether natural populations have the potential to adaptively respond to novel environmental stressors (Figure 1IA). Adaptive evolutionary changes may follow when genetic variants associated with an increased phenotypic ability to acquire material wealth increase in frequency in the population. As this process is highly environmentally dependent, we generally assume that selection via individual variation in material wealth promotes population adaptation to the local environment and to novel stressors. A striking recent example, the massive genome sequencing effort of Darwin's finches, shows that a few alleles with large effect on beak size changed in frequency across a 30‐year study period (Enbody et al. 2023). These variants affect individual fitness because beak size influences food competition outcomes and hence survival, especially during extreme climatic events.

BOX 1 **Material wealth**

In this study, we borrow the term ‘material wealth’ from the scientific literature on socioeconomics (e.g., Mulder et al. 2009), where material wealth is defined as ‘the abundance of valuable material possessions or resources’. From an evolutionary point of view, resources are valuable when they influence survival and reproduction. In pre‐industrial human societies material wealth was typically measured in terms of number of cattle owned or was based on land ownership, where the land owned varied in quality in terms of suitability for growing crops or for hunting. These material resources played a major role in ensuring a long lifespan and for the opportunity to raise large families (e.g., Skjærvø et al. 2011). The term ‘material wealth’ is therefore well‐suited to use when referring to an individual's ‘access to limited material resources needed for survival and reproduction’.

In modern humans, material wealth is comparatively easy to measure since money is used as a medium of exchange of material resources between individuals and money can also be used to measure the value of other assets such as estates owned. A wealthy person has access to a significant amount of money or owns possessions of significant monetary value. Measuring variation in material wealth among animals in natural populations is considerably more difficult. This requires in‐depth insights on the ecology and behaviour of the studied organism to identify the valuable material resources needed for survival and reproduction. It also requires very detailed studies and monitoring at the individual level to measure variation in access to these material resources. Since there is no equivalent for money, studies on natural populations need to be narrowed down to some component of material wealth with essential contribution to individual fitness. For example, a study on variation in material wealth among hermit crabs focused on access to shells that are needed for shelter and production against predators (Chase, Douady, and Padilla 2020). Our study on variation in material wealth among flycatchers focuses on access to caterpillars within the foraging range of breeding pairs, which is positively correlated with reproductive success (Rybinski et al. 2016; Sirkiä et al. 2018). Identifying the resources most essential to fitness and understanding variation in such material wealth across generations typically requires decades of monitoring, and demographic data such as the ones produced by the very long‐term studies highlighted in this special issue.

Improved local adaptation, whereby a population becomes genetically better adapted to the local environment (Williams 1966), is not the only possible evolutionary outcome of natural selection arising through individual variation in material wealth. Selection on heritable traits increasing the ability to acquire wealth enhances the competitive ability of local individuals compared to individuals from other populations. At the local population level, the outcome is not necessarily positive, as enhanced competitiveness of its members may undermine population growth and survival (‘tragedy of the commons’, Dieckmann and Ferrière 2004; Hardin 1968). The evolutionary implications of variation in material wealth largely depend on the underlying heritable phenotypic traits needed to acquire wealth (Figure 1IA). For example, evolution of phenotypic traits allowing more efficient resource exploitation generally have positive effects on the population, but evolution of phenotypic traits like aggressiveness, allowing individuals to monopolize resources, may undermine population growth (Baldauf, Engqvist, and Weissing 2014). Our ability to predict such different evolutionary implications is therefore limited by the fitness‐related traits in focus.

The inheritance of phenotypic traits related to an offspring's ability to acquire material wealth is not the whole story, as parents may affect it by additional means (Mousseau and Fox 1998; Rossiter 1996). Natal material wealth provided by the parents may have life‐long effects on their offspring's phenotype (e.g., body mass) affecting their ability to acquire material wealth later in life. In natural population studies, non‐genetic aspects of inheritance of material wealth are often referred to as ‘shared environmental effects’ or ‘silver‐spoon‐effects’, where the latter refers to high natal material wealth (Van de Pol et al. 2006). From an individual perspective, such effects are known to be important for fitness (Ilany, Holekamp, and Akçay 2021; Price and Boutin 1993; Smith 1968). In short‐lived animals, such non‐genetic aspects of inheritance of material wealth are generally assumed to be transient and most evident as cohort effects (Lindström 1999). To understand the evolution of ecologically relevant traits, non‐genetic shared environmental effects influencing parent‐offspring similarity in material wealth (i.e., Figure 1B,C) are therefore typically controlled for. This means that some fitness variation among family lineages is also controlled for, and that additional mechanisms causing similarity in environment (and in material wealth) among relatives remain unexplored. Moreover, when individuals sharing an environment also share genes, controlling for environmental effects means that the heritability of fitness‐related traits is underestimated because part of the genetic variation underlying both behavioural and morphological traits is partitioned as environmental effects (Gervais et al. 2022). This problem may be particularly relevant for phenotypic traits determining an individual's habitat choice.

Non‐genetic shared environmental effects on individual variation in material wealth may also include cultural transmission of behavioural traits such as habitat preferences (Mabry and Stamps 2008) or hunting skills (Foote et al. 2016, Figure 1IB). Most examples of culturally inherited material wealth come from long‐lived mammals with high cognitive abilities (e.g., African elephants (*Loxodonta africana*, Blumenbach 1797, Fishlock, Caldwell, and Lee 2016) and killer whales (*Orcinus orca*, Linnaeus 1758, Foote et al. 2016)). In these species, learned phenotypic skills associated with resource exploitation often play a central role in survival and reproduction (Figure 1IB). Since variance in fitness among family lineages is often difficult to measure in such long‐lived animals, it remains unknown whether cultural differences in resource use lead to variation in fitness that is consistent enough to cause genetic differentiation associated with resource quality. Non‐genetic inheritance of material wealth needs not always be indirectly mediated through inheritance of socially learned phenotypic abilities to acquire material wealth. Material wealth can also be directly transmitted from parents to offspring (Figure 1IC). Female red squirrels (*Sciurus vulgaris*, Linnaeus 1758) experience the advantage of reproducing earlier when they directly inherit a breeding territory rich in spruce cones from their mothers (Price and Boutin 1993; Smith 1968). Similarly, offspring may have priority to their parent's social rank and thereby experience high material wealth (Ilany, Holekamp, and Akçay 2021) instead of having to rely on inherited phenotypic traits to acquire it.

The different mechanisms of inheritance of material wealth (Figure 1IA–C) are not mutually exclusive but can have different evolutionary implications. Genetic drift may be reinforced if variation in material wealth largely depends on random events, for example, when a population colonises a new environment, and some individuals happen to find high‐quality, long‐lasting resources that become socially inherited (Figure 1B,C). These ‘lucky’ family members, experiencing enhanced reproductive performance thanks to high material wealth will then for several generations spread more genes. Hence, if non‐genetic mechanisms of inheritance of material wealth remain stable across generations evolutionary responses may be affected. An increased understanding of the drivers behind such evolutionary responses is highly relevant in the context of predicting whether and how natural populations can adapt quickly enough to anthropogenic stressors to avoid population decline and extinction. While non‐genetic inheritance of material wealth, especially direct inheritance (Figure 1IC), played a major role in the opportunity to raise large families in many pre‐industrial human societies (Skjærvø et al. 2011), the evolutionary importance of such drivers of variation in material wealth in natural populations remains largely unknown.

Studies of inheritance patterns, especially non‐genetic inheritance of material wealth, require long‐term monitoring of marked individuals. Here, we use a combination of genomic and phenotypic approaches to test whether material wealth is heritable and to investigate possible underlying mechanisms of inheritance (Figure 1IIA,B) in a monitored population of collared flycatchers (*Ficedula albicollis*, Temminck, 1815) from Öland, Sweden. This species has become a model system for studying ecological genomics (Qvarnström, Rice, and Ellegren 2010; Qvarnström et al. 2016), with a sequenced genome (Ellegren et al. 2012) and a 50 K SNP chip (Kawakami et al. 2014a). Collared flycatchers rely heavily on access to caterpillar larvae during the breeding season to ensure high reproductive success (Rybinski et al. 2016). We therefore use total caterpillar larvae biomass within the feeding range of breeding individuals as the currency of material wealth. We treat material wealth as an individual ‘state’ or ‘composite trait’ that can be heritable within a population in the same way as the heritability of specific fitness‐relevant phenotypic traits can be assessed (e.g., Kruuk et al. 2000). We find high genomic and quantitative genetic heritability of material wealth. These heritability estimates capture the combined additive genetic variance of all phenotypic traits influencing variation in the acquisition of material wealth (including non‐measured traits) in the population (Figure 1IIA). It also captures non‐causal genetic variation that may have become associated with various levels of material wealth through social inheritance patterns, as offspring inherit their parents' genes and material wealth in parallel (Figure 1IIB). Direct inheritance of material wealth is the simplest form of such parallel inheritance of genes and material wealth (Figure 1C) but is impossible in this case because flycatchers are migratory birds that establish new breeding territories each spring.

By using cross‐fostering experiments, long‐term monitoring data, gene set enrichment analyses and simulations we next investigate the importance of largely genetically determined phenotypic traits or learned traits (Figure 1IIA,B) in explaining the observed inheritance of material wealth. Based on the combined results, we conclude that the observed inheritance of material wealth largely arises as a consequence of a heterogeneous distribution of material wealth across the landscape in combination with strong philopatry, for example, due to a learned preference to return to the natal breeding site. This means that many (but not necessarily all) allelic variants associated with high material wealth in the population may lack causal effects on phenotypic traits that are used to acquire material wealth.

## Material and Methods

### Study Population

Collared flycatchers are small (12 g) passerine birds that spend the winter in Africa and breed in southern and eastern Europe. The first males arrive at the breeding grounds in late April and immediately start competing over natural breeding holes or nest‐boxes (Pärt and Qvarnström 1997). Females inspect several males before selecting their partner based on several characteristics of the male and the territory he defends (Dale and Slagsvold 1996). Juveniles fledge at 14–15 days old, are fed by parents for another week and remain in the vicinity for 3–4 weeks, ‘imprinting’ on the natal area until autumn migration in mid‐August (Löhrl 1959).

The areas of our study population on Öland, Sweden, include more than 2000 nest‐boxes, in deciduous, coniferous and mixed forest sites where we follow all breeding events since 2002. Briefly, all animals are ringed when first handled (either as 6‐day old nestlings, or breeding adults), blood is taken for later DNA extraction, and a variety of phenotypic traits are measured (see Qvarnström, Rice, and Ellegren 2010). Experimental procedures on collared flycatchers were approved by the Linköping Animal Care Board. The detailed population monitoring on Öland since 2002, allows for combining Genome‐Wide Association Study (GWAS) approaches with individual records of phenotypic traits and dispersal patterns.

### Individual Variation in Material Wealth

Flycatchers feed their offspring up to 80% nutrient‐rich caterpillars, which increases fledgling mass and survival (Burger et al. 2012; Cramp and Simmons 2006; Eeva et al. 2010; Linden, Gustafsson, and Part 1992). Material wealth, the biomass of herbivorous *Lepidoptera* and *Hymenoptera* larvae in the birds' breeding territories, was estimated from measures of tree‐specific caterpillar abundance, tree density and volume (see supplementary information, Rybinski et al. 2016). Individual between‐years repeatability was calculated using ‘rptR’ (Stoffel, Nakagawa, and Schielzeth 2017) with a Gaussian distribution and 1000 bootstrap interactions.

### Genome‐Wide Association Study

We genotyped 825 collared flycatchers on a 50 K SNP array (Kawakami et al. 2014a), and pruned for linkage disequilibrium (threshold: *r* ^2^ = 0.7), leaving 36,773 SNPs for 800 individuals (414 males, 386 females, see Supplementary Methods, Husby et al. 2015). We quantified the relationship between genetic variation and material wealth using a GWAS, fitting material wealth as the response variable, with SNP effects, genetic relatedness between individuals (GRM, calculated from the genotypes by ReapeatABEL, Rönnegård et al. 2016), sex and year as fixed effects and individual as a random effect.

We calculated the *p*‐value of each SNP effect by computing a standard Wald statistic, based on the estimated SNP effect and its standard error. Using a Bonferroni correction, the genome‐wide significance threshold is *p* = 1.29 × 10^−6^ (Figure 3A, top dotted line). Any SNPs with lower *p*‐values than this threshold were considered significant, although given that we expect a polygenic trait, or, indeed that significant SNPs given our sample size are likely to have overestimated effect sizes (‘Beavis effect’, Beavis 1994, 1998; Josephs, Stinchcombe, and Wright 2017; Slate 2013), we were interested in the rank order of all SNPs and assumed that our parameter estimates for any significant SNPs could be upwardly biased (‘winner's curse’, Palmer, and Pe'er, I. 2017), while also being concerned about the potential for overfitting leading to limited generalisability and predictive power (James et al. 2021; Marigorta et al. 2018). For these reasons, in downstream analyses, we always considered the top 100 SNPs (with the lowest *p*‐values), rather than differentiating between significant and non‐significant SNPs.

### Animal Model

We decomposed variance using mixed effects animal models using the resemblance between related individuals to determine the additive genetic variance and genetic correlations of material wealth (Kruuk 2004). We used a pedigree from 2002 until 2020 with 33,384 individuals, pruned down to the 5644 informative individuals (3054 females) for which we had material wealth measurements using prunePed (Morrissey and Wilson 2010) in MCMCglmm (Hadfield 2010). Note that most individuals in the pedigree are nestlings which did not recruit. We fit a univariate animal model to material wealth, with additive genetic variation (*h* ^2^), permanent environmental effect (pe^2^) and year (year^2^) as random effects.

### Cross‐Fostering Experiment

To disentangle genetic and non‐genetic inheritance mechanisms of material wealth we used cross‐fostering experiments. Complete clutches of eggs were exchanged between nests in 2002, 2007, 2008, 2009 and 2017 (reciprocal cross‐fostering), and partial cross‐fostering (two to four individuals exchanged between clutches) of 3 days old nestlings was done from 2010 to 2022. We used nests with similar clutch sizes and identical hatching date, to minimise strong size differences between half‐siblings (see McFarlane et al. 2018; Vallin et al. 2013). If variation in material wealth is determined by heritable phenotypic abilities to acquire resources, we expect recruiting nestlings to return to breed in a territory with material wealth more similar to their genetic than to their foster parents. In total, 53 adults (46% females) recruited from these cross‐fostering experiments and bred in our population between 2003 and 2022. The difference of material wealth between their genetic and foster parents varied between 0 and 1.65 (mean = 0.27), covering almost the entire variation in our population. We used a linear mixed model to compare material wealth of their first breeding territory to both the material wealth of their foster parents (where they were raised), and that of their genetic parents, with genetic and foster nests as random effects.

### Phenotypic Traits and Material Wealth

To test if measured heritable phenotypic traits underlie inheritance of material wealth (Figure 1IIA), we used a linear mixed effect model to investigate possible associations between five ecologically important and/or known fitness‐related traits (forehead patch size, wing length, tarsus length, mass, laying date) and material wealth of 1103 adult birds (561 females) born in our population that returned to breed between 2002 and 2020. Forehead patch size signals fighting ability in territorial disputes (Pärt and Qvarnström 1997), tarsus and mass represent size (bigger individuals may be more competitive), wing length affects manoeuvrability (important in aerial fights) and laying date (standardised around each year's peak of laying) is a proxy for arrival date at the breeding ground (which influences availability of unoccupied territories). We also tested if condition near fledging influences material wealth as an adult (potential silver spoon effect) by including each adult's own earlier mass measured when they were 12 days old. Material wealth of the first recorded breeding territory was fitted as a response variable with the aforementioned heritable phenotypic traits (all centred around the mean), age and sex as fixed effects, and year, family ID (accounting for returning siblings) and measurer as random effects (see Table 1 for variance inflation factors). We tested for genetic correlations between material wealth and forehead patch size, laying date, hatching and fledging success using bivariate animal models, and for genomic associations between top material wealth SNPS, forehead patch size and laying date using LASSO analyses (Supplementary Methods).

**TABLE 1:** Results of a linear mixed model testing for correlations between material wealth of the first breeding territory, five adult phenotypic traits (forehead patch, wing length, tarsus length, mass and age) a nestling phenotypic traits (mass at 12 days of age of the same individual in its parent's nest) as well as a behavioural trait (laying date, proxy for timing of arrival on the breeding grounds) and sex on 1003 individuals born in our monitored population.

Fixed effects	VIF	Material wealth and phenotypic traits			
		Estimate	SD	*t*‐value	*p*
Intercept	—	1.41	0.12	11.63	**<0.001**
Forehead patch	1.24	−0.00	0.00	−0.73	0.465
Wing	1.11	−0.00	0.01	−0.12	0.906
Tarsus	1.13	0.00	0.02	0.10	0.924
Adult mass	1.45	0.02	0.02	0.62	0.533
Age	1.10	−0.03	0.03	−1.06	0.288
Relative laying date	1.03	−0.00	0.00	−0.11	0.911
Mass as 12 days old young	1.17	0.01	0.02	0.71	0.481
Sex	1.46	−0.11	0.13	−0.83	0.410

Random effects	Variance	*N*
Natal nest ID	0.09	337
Measurer	0.01	28
Year	0.00	18

*Note:* We tested for multicollinearity between the variables and report variance inflation factors (VIFs) in the first column. The condition number (κ) of the correlation matrix for this analysis was 17.45. Significant *p* values (*p* < 0.05) are shown in bold.

### Gene Set Over‐Representation Analysis

To explore the functions of genes underlying the genomic heritability of material wealth, genes were extracted in windows 100,000 bp upstream and downstream of the 100 top material wealth SNPs (433 genes). We used clusterProfiler (Wu et al. 2021) to identify gene sets over‐represented among the 274 annotated material wealth genes compared to all functions found in the collared flycatcher genome (10,610 genes with gene set information, Ensembl release FicAlb1.5, Kawakami et al. 2014b). *p* values were adjusted using Bonferroni correction.

### Inheritance of Material Wealth Through Learned Phenotypic Wealth Acquisition Abilities

Traits that are mostly non‐genetically inherited through learning such as preferences for the natal site (causing natal philopatry) or preferences for specific characteristics of the natal site are likely to influence the similarity between natal and adult material wealth (Figure 1IIB). We examined the effect of natal dispersal distances on inheritance of material wealth of 1007 birds for which we had measured both their natal and first breeding material wealth. We used global positioning devices to obtain exact coordinates for these birds' nestboxes. We calculated Euclidian distances between the natal nest (where a bird was ringed as juvenile) and the first breeding location of this same bird as adult. To test if the relationship between natal and first breeding territory's material wealth depends on dispersal distance we used a linear mixed model with breeding material wealth as response variable and natal material wealth, dispersal distance and the interaction between them as fixed effects (because material wealth may influence dispersal distance, see below), with birth nest as a random effect.

Since adaptive dispersal decisions may depend on the conditions experienced as young, we also investigated whether natal material wealth influenced subsequent dispersal in a linear mixed model with natal dispersal distance (distance between birth nest and first breeding nest) as response variable, natal material wealth as explanatory variable and birth nest as a random effect. We used a simulation to investigate if the observed relationship between natal and first breeding material wealth could be explained by spatial autocorrelation in material wealth of nearby breeding territories (Data S1).

All analyses were done in R (versions 4.1.2 and 4.3.1). All code and data are available on Dryad. doi:10.5061/dryad.dz08kps4s

## Results

### High Heritability of Material Wealth

**FIGURE 2:**
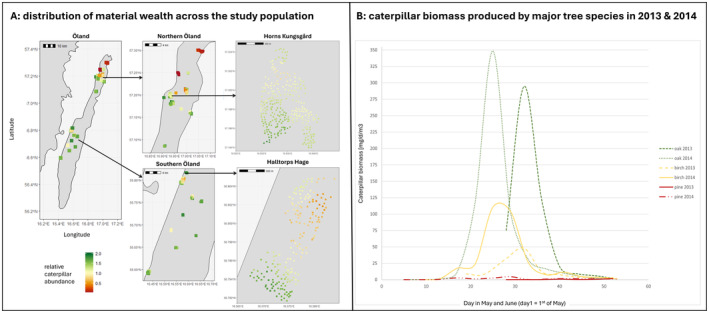
(A) Illustration of the distribution of material wealth (estimated as caterpillar larvae biomass) among breeding territories across the whole island of Öland, the Northern and Southern study areas and the two largest monitored forest patches. Individual squares in the third panel represent individual feeding areas in a 150 m radius around each nestbox. (B) Caterpillar larvae biomass (i.e., estimated from caterpillar faeces or frass) produced across three common tree species (representing rich, medium and poor habitats) in 2013 and 2014.

**FIGURE 3:**
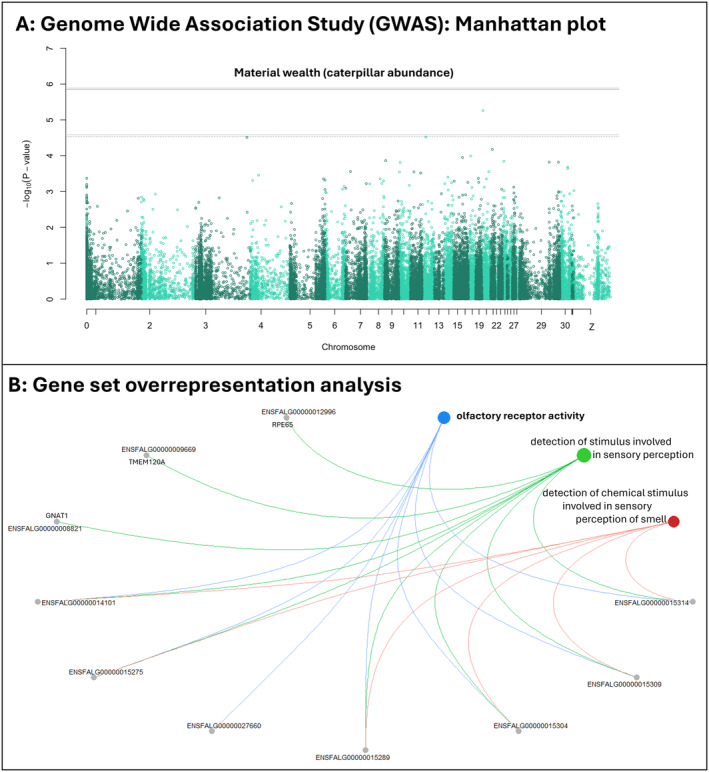
(A) Manhattan plot representing the Genome Wide Association between genetic variants and material wealth. The *y*‐axis represents the –log 10 *p*‐values from the repeated measures screen for the association between marker genotype and material wealth for all 38,705 SNPs along the chromosomes (*x*‐axis). The top line indicates the genome‐wide Bonferroni‐corrected significant threshold and the lower line the threshold for suggestive significant associations. (B) Gene set significantly over‐represented among the genes associated with the top 100 SNPs from the GWAS analysis of material wealth and the genes and related pathways linked to it.

Individual territories occupied by collared flycatchers in 2002–2020 had values of material wealth between 0.14 and 2.03 (mean = 1.31 ± 0.35, Figure 2A, Figures S1 and S2) and the between‐year repeatability of material wealth for an individual bird was 0.74 (±0.008). Based on 36,773 SNPs distributed across the collared flycatcher genome, we find a high genomic heritability of material wealth (*h* ^2^ = 0.405 ± 0.08) in terms of caterpillar larvae biomass (frass/m^2^) available within their breeding location's feeding range. No single SNP significantly explained material wealth (Figure 3A). An animal breeding model relying on the whole pedigree reveals a quantitative genetic heritability of material wealth (*h* ^2^) of 0.232 (95% CI: 0.16–0.29, Table S1).

### Disentangling Genetic and Non‐Genetic Mechanisms of Inheritance of Material Wealth

**FIGURE 4:**
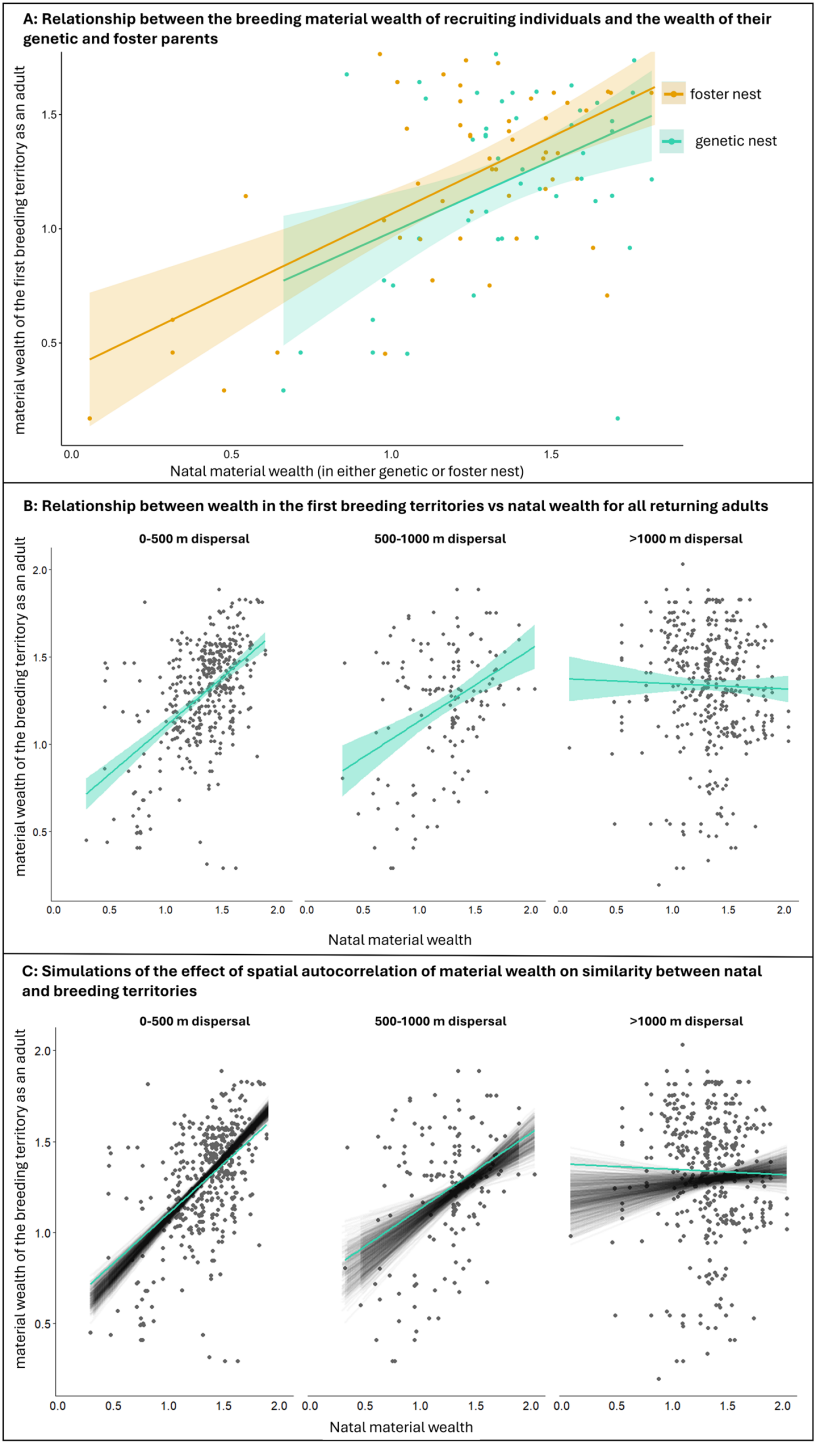
(A) Relationship between the breeding material wealth (mass of caterpillar faeces/m^3^) of 53 recruiting individuals that were subjected to a cross‐fostering experiment as young (*x*‐axis) and the material wealth of their genetic (blue‐green, lower slope) and foster (yellow, upper slope) parents. (B) Relationship between material wealth in the first breeding territories versus natal material wealth for 1007 returning adults born in our population, depicted at three categories of dispersal distance: Short and medium, as well as long dispersal distances. Note that the correlation between natal and breeding material wealth disappears at long dispersal distances. (C) Simulations of the effect of spatial autocorrelation of material wealth on similarity between natal and breeding territories. Each black line represents one of 500 simulations, plotted on top of the original datapoints (dark grey) and empirical correlation (blue‐green line) between natal and breeding material wealth shown in panel (B).

We compared the material wealth of 53 recruits that had been cross‐fostered to their foster and genetic parents' material wealth. There was a significant effect of the foster parents' ($\chi_{1}^{2}=18.9$, *p* < 0.001), but not of the genetic parents' material wealth ($\chi_{1}^{2}=2.21$, *p* = 0.137, Figure 4A, Table S2), on the recruits' material wealth. Cross‐fostering experiments are constrained by the need to match similar nests to avoid biased sibling competition and we can only measure material wealth in the subset of nestlings that survive and return to breed. Genetic and foster parents' material wealth was therefore positively correlated (*r* = 0.43) and there was no significant difference in the slope of the association between foster or genetic nest and material wealth of returning adults (estimates = 0.67 ± 0.13 and 0.58 ± 0.2, respectively, *p* = 0.72). Thus, although these results are indicative of an important non‐genetic component of inheritance of material wealth (Figure 1IIB), the experimental design does not allow ruling out a potential effect of genetically inherited phenotypic traits.

### Do Allelic Variants Associated With Material Wealth Encode Genetically Inherited Phenotypic Traits?

To investigate the role of well‐known genetically inherited fitness‐related traits in explaining the inheritance of material wealth (Figure 1IIA), we tested if these traits predicted their bearer's material wealth. None of these traits were significantly correlated with material wealth (*p*‐values between 0.29 and 0.92, see Table 1), although we should keep in mind that any effect of small magnitude may have been masked by the existence of correlations between some of the traits tested. Similarly, none of the tested traits were genetically correlated with material wealth (forehead patch: corr = −0.021, 95% CI −0.13 to 0.10, laying date: corr = 0.011, 95% CI: −0.53 to 0.73, see Table S3 for all traits). Based on previous knowledge of the system, we also investigated if the 100 SNPs most strongly associated with material wealth (Figure 3A, Table S4) were associated with two particularly interesting candidate material wealth acquisition traits: forehead patch size (that predicts male competitive ability) or laying date (a proxy for the timing of territory establishment). We found no evidence for such associations: none of the SNPs were retained by either LASSO model (parameters not estimated). Together, these findings imply that the observed heritability of material wealth is largely explained by either (a) unmeasured genetically inherited phenotypic traits linked to material wealth acquisition (Figure 1IIA), or (b) mostly non‐genetically inherited phenotypic wealth acquisition traits such as a learned preference for the natal site (Figure 1IIC). We therefore tested for functions over‐represented among the genes linked to these top SNPs associated with material wealth compared to the whole genome to detect possible unmeasured genetically inherited material wealth acquisition traits. One gene set was significantly over‐represented: ‘olfactory receptor activity’ (Gene Ratio = 7/298, *p*_adjusted_ = 0.034, Figure 3B, Table S5).

### Inheritance of Material Wealth Through Learned Phenotypic Wealth Acquisition Abilities

To investigate the potential role of (mostly) learned phenotypic wealth acquisition traits (Figure 1IIB), we used 1007 collared flycatchers measured as nestlings that returned to breed to examine the effect of natal dispersal on similarity in material wealth among relatives. Birds on average dispersed 1272 ± 1382 m from their natal nest. Dispersal distance was not affected by natal material wealth (*F*_1,754_ = 1.455, *p* = 0.228). However, natal material wealth interacted with dispersal distance to explain the first breeding territory's material wealth (*F*_1,988_ = 14.343, *p* < 0.001, Table S6). Birds that dispersed shorter distances (<1000 m, Figure 4B) from their natal nest experienced a higher parent–offspring similarity in material wealth than did birds dispersing longer distances (>1000 m, Figure 4B). Simulations suggest that spatial autocorrelation in material wealth across the landscape underlies this pattern (Figure 4C). Thus, parent–offspring similarity in material wealth is mainly caused by philopatry per se rather than by habitat imprinting.

## Discussion

Our study reveals large variation in material wealth of individual collared flycatchers (Figure 2) and that material wealth as a state/ composite trait is inherited. We report a significant pedigree‐estimated quantitative genetic heritability of material wealth (*h* ^2^ = 0.2), indicating that offspring resemble their genetic parents in material wealth. We find an even higher genomic heritability of material wealth (*h* ^2^ ~ 0.4), indicating that variation in material wealth is to a considerable extent explained by variation in genomic markers. As our measure of material wealth is known to predict reproductive performance, an important component of fitness (Sirkiä et al. 2018), one would expect the allelic variants associated with high material wealth to increase in frequency in the population. Important questions then follow: what biological functions are these allelic variants coding for and through which inheritance mechanisms do they become associated with material wealth (Figure 1IIA or B)?

We found little evidence suggesting that allelic variants associated with material wealth encoded variation in phenotypic characteristics that we expected to influence the acquisition of this important resource (see Supplementary Discussion). However, gene set enrichment analyses of the top SNPs associated with material wealth reveal a significant over‐representation of olfactory pathways. Olfaction plays an important role in homing behaviour in several wild bird species (Abankwah, Deeming, and Pike 2020). There is also experimental evidence suggesting that great tits (*Parus major*, Linnaeus 1758) use scent to relocate to feeding sites after displacement (Mahr et al. 2022) and can respond to herbivore‐induced plant volatiles (Delaitre et al. 2024). Whether olfaction plays a similar role in collared flycatchers remains unknown, making it premature to conclude a causal relationship between variation in olfaction alleles and acquisition of material wealth in terms of caterpillar biomass in the breeding territory.

Similarities in material wealth among genetic relatives can arise through mechanisms other than underlying heritable phenotypic abilities to acquire wealth. To disentangle the main mechanisms for material wealth inheritance (Figure 1IIA,B) we used cross‐fostering experiments, analyses of dispersal patterns and simulations. We found a strong resemblance in material wealth between foster parents and their foster offspring, indicating that a non‐genetic mechanism of inheritance is important. Detailed information about natal dispersal patterns obtained from our long‐term breeding records reveals that short natal dispersal distance (high philopatry) is a prerequisite for the observed association between genetic variants and material wealth (Figure 4). There is no similarity in material wealth between parents and their offspring in cases where offspring breed far away from their natal site (Figure 4B), implying that natal philopatry per se rather than habitat imprinting explains heritability of material wealth. This is because we would not expect an effect of dispersal distance if parent‐offspring similarity in material wealth had resulted from imprinted habitat preferences, as the same type of habitat is scattered across our study plots (Figure 2A). Our results are hence most consistent with the inheritance of material wealth in collared flycatchers being largely driven by natal philopatry in a heterogeneous environment, which is also supported by the simulations (Figure 4C). Philopatry thus induces population structure, where members of the same family lineages cluster spatially, resulting in high genomic heritability of material wealth. These associations between allelic variants and material wealth are ‘spurious’, that is, relatively short‐lived and expected to decay gradually over several generations. This decay is due to variation in dispersal distances in the population and because the distribution of material wealth across the landscape will eventually change over longer time scales (see Supplementary Information for an in‐depth discussion on resource distribution consistency).

Since resources often are unevenly distributed across space and individuals settling first in an area generally have a competitive advantage (Kokko, López‐Sepulcre, and Morrell 2006), associations between genetic variance in a population and variation in material wealth may also in other cases arise because of philopatry. Despite a general agreement on the importance of site‐knowledge in determining breeding settlement patterns in many animal species with high dispersal ability (e.g., Greenwood 1980), its potential to cause similarity in material wealth between parents and offspring across several generations and result in high heritability of material wealth has to our knowledge not previously been investigated.

From a theoretical perspective a constant level of very high philopatry is unlikely to be evolutionary stable because individuals born on a productive site profit from philopatry, while individuals born on an unproductive site should disperse. We therefore also expected a negative relationship between material wealth and natal dispersal distance in flycatchers, but we found no such relationship. Possible explanations for the absence of negative relationship include higher competition for breeding territories in wealthy areas (some individuals born on wealthy sites may be ‘forced’ to settle elsewhere). Additionally, the benefits of local familiarity may favour philopatry even for individuals born in poorer habitats. We would like to encourage future studies on other species to evaluate the generality of philopatry‐driven inheritance of material wealth.

Previous studies on inheritance of breeding sites in birds have focused on territory inheritance to explain the evolution of cooperative breeding (Koenig et al. 2023; Lindström 1986; Stacey and Ligon 1991). However, these studies were generally not aimed at investigating possible patterns of long‐lasting material wealth inequality among family lineages resulting in material wealth becoming a heritable ‘state’. A study on Darwin's Medium Ground Finches (*Geospiza fortis*, Gould 1837), the only one we are aware of that investigated the heritability of material wealth, revealed a significant heritability of territory size (Price 1984). This was at least partly explained by variation in body size and interpreted as a silver spoon effect mediated by condition, where offspring raised on large territories grew bigger and were therefore later able to defend larger territories.

Variation in material wealth is an important source of natural selection but in many group‐living, social animals (especially humans), non‐genetic inheritance patterns may cause fairly long‐lasting biases in material wealth among families that may be decoupled from underlying genetically inherited morphological, behavioural or cognitive abilities (e.g., Fishlock, Caldwell, and Lee 2016; Foote et al. 2016; Skjærvø et al. 2011). Our study illustrates that philopatry can cause inheritance of material wealth also in a short‐lived species lacking complex social structures. Philopatry‐induced heritability of material wealth may, in general, have consequences for a population's ability to respond to environmental stressors. Adaptive evolutionary change restoring positive growth of declining populations under environmental change (‘evolutionary rescue’, Carlson, Cunningham, and Westley 2014) may indeed be hindered or delayed in this scenario. This is because philopatry causes an association between genotypes and material wealth where many allelic variants associated with high material wealth may lack causal connections to ecologically relevant traits of importance to track environmental changes. This is a situation that may reduce the efficiency by which natural selection promotes adaptive changes and may even favour the spread of mildly deleterious alleles if such mutations arise in family lineages experiencing high material wealth simply by tradition. However, there are also counterarguments. A genetically structured population typically ‘stores’ more genetic variation, allowing a more rapid response to novel challenges. Fast deterioration of the local environment could also induce higher dispersal once local food availability has reached a particularly low threshold value, whereby colonisation of novel breeding sites could reduce the risk of population decline by facilitating ‘niche tracking’ (Fandos et al. 2020). Modelling the overall impact of philopatry on population growth and evolvability would be useful to evaluate the likelihood of these different scenarios.

Our study illustrates the importance of using long‐term data on known individuals to reveal mechanisms of inheritance of material wealth and for robust interpretation of a population's genetic structure across the landscape and in relation to the distribution of key resources needed for reproduction. We encourage future theoretical and empirical studies evaluating the importance of non‐genetic inheritance of material wealth for population dynamics and evolutionary trajectories also in short‐lived species lacking complex social structures.

## Author Contributions

M.Å., S.E.M. and A.Q. designed the study with contributions from T.P. and F.J.W. M.Å., S.E.M., A.H., P.S., D.W., Y.Z. and A.Q. collected data. M.Å., S.E.M. and J.K. performed data analyses. A.Q. wrote the manuscript. All authors contributed to interpretation of results and commented on the manuscript.

## Conflicts of Interest

The authors declare no conflicts of interest.

### Peer Review

The peer review history for this article is available at https://www.webofscience.com/api/gateway/wos/peer‐review/10.1111/ele.14505.

## Supporting information




Data S1.

## Data Availability

The data and code supporting the results are archived at Dryad. doi:10.5061/dryad.dz08kps4s.
