# Supplementary material for: Inheritance of Material Wealth in a Natural Population
Source: Ecol Lett. 2024 Dec 31;27(12):e14505. doi: 10.1111/ele.14505 (PMC11686942; doi:10.1111/ele.14505)
Supplement: Supplementary file 1 — Data S1. [file ELE-27-0-s001.docx]

**This is the Supporting Information corresponding to the manuscript “Inheritance of material wealth in a natural population”.**

**Abstract**

**Evolutionary adaptation occurs when individuals vary in access to fitness-relevant resources and these differences in “material wealth” are heritable. It is typically assumed that the inheritance of material wealth reflects heritable variation in the phenotypic abilities needed to acquire material wealth. We scrutinize this assumption by investigating additional mechanisms underlying the inheritance of material wealth in collared flycatchers. A genome-wide-association analysis reveals a high genomic heritability (h^2^=0.405+/-0.08) of access to caterpillar larvae, a fitness-relevant resource, in the birds’ breeding territories. However, we find little evidence for heritable variation in phenotypic abilities needed to acquire this material wealth. Instead, combined evidence from simulations, experimental and long-term monitoring data indicate that inheritance of material wealth is largely explained by philopatry causing a within-population genetic structure across a heterogeneous landscape. Therefore, allelic variants associated with high material wealth may spread in the population without having causal connections to traits promoting local adaptation.**

**Table of contents:**

Supporting methods on material wealth:

Estimate and stability of material wealth during breeding

Genome Wide Association Study

Estimation of genetic correlations based on pedigree data and genomic markers

Simulations of dispersal distances across the observed distribution of material wealth

Supporting discussion

Supplementary figure S1-S2

Supplementary tables S1- S6

Supplementary references

***Estimating material wealth during the breeding season***

We focus on food availability in the breeding territories of collared flycatcher pairs as our estimate of material wealth. Food availability is tightly associated with reproductive performance in birds (Arnold et al., 2010; Siikamäki Pirkko, 1998; Slagsvold & Wiebe, 2007) and herbivorous *Lepidoptera* and *Hymenoptera* larvae (hereafter caterpillars) represent a nutrient-rich, high-quality food resource for insectivorous birds, such as collared flycatchers (Burger et al., 2012). Every year, collared flycatcher migrates all the way from Africa to use this ephemeral but at the same time predictable food resource. Migration is an energetically costly and dangerous behavior associated with high mortality and yet undertaken by many species of birds. This costly behavior is driven by temporal availability of food. A high proportion of caterpillars in the diet increases fledgling mass, and ultimately, increases the likelihood of offspring survival (Burger et al. 2012, Linden et al. 1992). Collared flycatchers therefore need to time their breeding to match the peak in their offspring’s growth curve to the peak in caterpillar abundance. Both breeding too early or too late are associated with strongly reduced breeding performance in our study population (Sirkiä et al., 2018), and we therefore estimated caterpillar biomass during the breeding season. This data was produced and used in two previous studies: (Rybinski et al., 2016; Sirkiä et al., 2018). In short, we obtained our estimate of material wealth by using the following steps. 1) We measured caterpillar biomass associated with the 11 most common tree species occurring at the breeding sites, and 2) combined this measure with nest-box point estimates of habitat tree species composition and volume to estimate food-availability within short ranges of the nest-boxes, and finally 3) we extrapolated the point data to estimates caterpillar biomass within the typical foraging range of 150 m of each breeding pair (see details below).

1) Measure of caterpillar biomass associated with the 11 most common tree species occurring at the breeding sites: The availability and seasonal pattern of caterpillar larvae differ between tree species, but this knowledge was, before our studies, limited to a few studied tree species (Nadolski et al., 2021; Van Asch & Visser, 2007; Veen et al., 2010). Temperate forests are however diverse in their tree species composition and we collected “frass” fall (i.e. fecal droppings from leaf eating caterpillar larvae) from the 11 most common tree species in our study areas in 2013-2014. Estimation of caterpillar biomass was quantified by the amount of frass, a commonly used method in ecological studies of birds (e.g. Van Balen 1973; Visser et al. 2006). Larvae pellets were collected by custom-built traps that consisted of 50cm x 50cm wooden frames that were positioned 50 cm above the ground and about 1m from the tree trunk. For the most common tree species (hazel, *Corylus avellana*; oak, *Quercus robur*; ash, *Fraxinus excelsior*; birch, *Betula* sp.; pine, *Pinus* *sylvestris*; alder *Alnus* sp.; elm, *Ulmus* sp.; European hornbeam, *Carpinus betulus*; spruce, *Picea abies*; lind, *Tilia cordata*; maple, *Acer platanoides*) two traps per tree were used. The selected tree species represented 97 % of all the tree species present in the study areas. In 2013, 41 individual trees were sampled for frass. In 2014 we repeated the sampling and 38 out of 41 frass traps were located under the same trees in both study years. In addition, we sampled 10 extra individual trees in 2014 (hazel, oak, birch, pine, spruce). The frass sampling started soon after the bud burst of the earliest tree species and lasted until the end of the flycatcher breeding season in 2013 and 2014. The nets from the traps were replaced every 4th day during the season. Collected nets with frass inside were dried indoors and afterwards frass was separated from the coarse fraction of the litter with dense standard sized sieves. The samples were stored frozen. After the field season the remaining litter was separated manually in the laboratory and the amount of dry frass was weighed to an accuracy of 0.01 mg. Because the amount frass depends on the tree crown height above the trap, we measured the crown height above each trap using a clinometer (Suunto PM-5/400 PC) to convert the dry mass of frass from each trap to mg frass/day/m^3^ canopy measured. We then calculated the sum of the mean daily frass values (i.e. frass/day/m^3^ canopy) from all traps placed under individual trees belonging to the same tree species. In this way, we obtained tree-species specific values of caterpillar abundance during the nestling phase of the flycatcher breeding cycle.

We found large differences in amount of frass among the tree species with the highest peak in frass collected from hazel, oak and lime (well above 30 frass mg/m^3^ tree/day), while the values for pine and spruce never reached above 10 frass mg/m^3^ tree/day (Figure S1). The sampling period covered 80% of the nestling phase of the flycatcher breeding cycle, as averaged from the whole 14-year dataset collected before the frass sampling years. In the analyses we used the mean frass values of two sampling years. There are some differences in the timing of the peaks in caterpillar larvae biomass between study years (main text figure 2B), but flycatchers adjust their timing of breeding accordingly (Sirkiä et al 2018). The large relative differences in caterpillar biomass between tree species remain very constant between years because caterpillars are co-adapted to their host species and therefore have clear host tree species preferences. Individual birds that arrive late at the breeding grounds may make the best-of-a-bad-job by breeding in mixed-forest sites with a later peak in caterpillar biomass (Veen et al 2010). However, the reproductive performance of such late breeding birds is lower than the reproductive performance of the early breeding birds that managed to benefit from the high caterpillar abundance in some of the deciduous tree species such as hazel, oak and lime (Rybinski et al 2016).

2) Point estimates of food availability around specific nest-boxes. Tree species composition was measured around specific nest-boxes with a relascope (Bitterlich, 1984), which is a commonly used variable radius sampling method used in forestry and ecological studies to quantify the amount of wood in the forest (Huhta et al., 1998; Pommerening & Sterba, 2024). We calculated the basal area of different tree species around nest-boxes based on the relative size of the tree trunks assessed from a single point next to a nest-box. The method accounts for both the tree trunk size and the distance from the observer. Each tree trunk was classified into one of three categories and assigned a value; (A) small (value 0); (B) medium sized (value 0.5) and (C) large (value 1). For birch (*Betula* spp.), alder (*Alnus* spp.), elm (*Ulmus* spp.) and willow (*Salix* spp), the counts were done on the level of the genus and 19 tree taxa were recorded in total. A mature hazel bush, typically consisting of several thin trunks close together, was considered as one tree trunk. For each nest-box point estimate of habitat composition all basal area values per tree species were summed. These point estimates of habitat composition and volume were obtained for 421 different nest-boxes evenly spread across our monitored breeding sites and, on average, four of the considered tree species were present within these points (mean 4.44 ± 0.15) with a range from 1 to 8 tree species. There was considerable variation in the relative abundances of the different tree species, ranging from an average of 0.4 individual trees (alder, spruce) to 16.1 (hazel). We then combined the mean caterpillar biomass data for each tree species (see 1 above) with these nest-box point estimates of habitat tree species composition and relative volume to obtain an estimate of food availability during the flycatchers’ nestling phase for each specific nest-box sampling point. These point estimates of food availability thus combine the caterpillar biomass production associated with particular tree species with the relative number and volume of these tree species around a particular flycatcher nest-box sampled.

3) Material wealth of breeding flycatcher pairs: Since the foraging ranges of flycatchers extend into neighboring territories, we consecutively calculated the average caterpillar abundance of all nest-box point estimates obtained within a radius of 150m from each breeding flycatcher pair. We hence used the acquired 421 point estimates of food availability (resulting from combining 1 and 2 above) to calculate the expected caterpillar biomass production within the foraging ranges used by breeding flycatcher pairs. Our estimate thereby combines the information about caterpillar biomass production associated with particular tree species with the relative densities and canopy coverage of the trees within a radius of 150 m from the focal nest. This extrapolation was based on GPS coordinates of all the nest boxes in our dataset. First, 150m radius zones were created around each nestbox that was occupied by a breeding flycatcher pair. Then “Point statistics for polygons” SAGA algorithm (Cimmery, 2007; Olaya, 2004) was used to obtain averaged values of caterpillar biomass production. The algorithm used two layers: point layer – which was the layer containing point estimates of food availability; and polygon layer – which contained the feeding range zones around each breeding pair from our dataset. The point estimates of food availability overlapping with each polygon were used to calculate the extrapolated values for the foraging range of flycatcher pair the polygon represented. For the sake of the algorithm used, the point estimate of food availability was represented as point data, however it is important to remember about its true, area like, nature (described above). Even though in the algorithm the data is associated with a fixed point in space, it still represents the average food availability based on the composition of the habitat around the focal nest-box. Thus, our measure of material wealth is an estimated amount of caterpillar larvae biomass available within a 150 range from the occupied nest-box.

***Stability in relative material wealth across breeding seasons***

Tree species composition and volume have remained relatively stable during the last twenty years even if some individual trees died. Most of our study sites are located in natural reserves and with no clear cutting of trees. Some of the trees, like the caterpillar rich oaks are several hundreds of years old, which translates into very many flycatcher generations. Availability of caterpillars may vary across years due to e.g. temperature and rainfall but the relative production between feeding territories is likely remaining the same, due to the herbivorous *Lepidoptera* and *Hymenoptera* having clear tree species preferences. In a short evolutionary perspective, which is relevant in the context of evolutionary responses to fast changes in the environment, we expect the documented large effect of site fidelity on inheritance of material wealth to play a major role. This is because the high genomic heritability of material wealth means that there are associations between allelic variants and material wealth, which in turn correlated positively with fitness.

***Genome Wide Association Study***

In the GWAS analyses, we excluded individuals or markers with <95% call rate, those markers with minor allele frequency of <0.01 and a Hardy-Weinberg equilibrium (HWE) p-value <0.001 using PLINK (Purcell et al., 2007) and the check.marker command in GenABEL (Aulchenko et al., 2007), as done in Silva et al. (2017). To take advantage of the individuals for which we had repeated breeding records, we used the package RepeatABEL to perform GWAS on the repeated samplings of related individuals (Rönnegård et al., 2016). RepeatABEL fixes the SNP effects by using a linear mixed model, including permanent environmental effects and random polygenic effects with correlation matrices given by the genomic kinship matrix (Rönnegård et al., 2016). RepeatABEL has been shown to increase the power in wild system GWAS, particularly in variable environments (Rönnegård et al., 2016).

***Estimation of genetic correlations based on pedigree data and genomic markers***

To estimate genetic correlations between material wealth and each forehead patch size (important for territory access), and laying date, hatching success and fledging success (important fitness traits), we fit bivariate animal models (see general methods for animal models above), where genetic covariance was fit (’us’) but permanent environmental effects were assumed to be independent for each trait (’idh’) (Hadfield, 2010). Material wealth, forehead patch size and year-standardize laying date (where the mean lay date for the year was subtracted from the lay date) were fit as ’gaussian’ response variables, while the number of hatched and fledged nestlings were fit as ’poisson’ response variables. We used parameter expanded, non-informative priors (Hadfield, 2010), and report the posterior modes and 95% confidence intervals of all estimated variance components. All animal models were run for at least 500,000 iterations, with 100,000 iterations of burn-in, with thinning as needed to sample 1,000 points. Some models were run for longer (up to 15,000,000 iterations) to achieve convergence, which was tested by assessing the autocorrelation as well as Heidelberger & Welch (1983) and Geweke (1991) convergence diagnostics.

We used a LASSO (least absolute shrinkage and selection operator) to apply a sparse modelling approach to examine if any of SNPs most highly associated with material wealth were also associated with phenotypic traits. LASSO can be used for variable selection and parameter estimation when n<<p (where n is the number of individuals and p is the number of parameters), as the model uses penalization via shrinkage to avoid overfitting. In this way, parameter estimates can be shrunk to zero and not estimated (Hastie et al., 2009). Here, we used the package glmnet and implemented a cross validation to determine the appropriate penalization (lambda) to fit for each regression (Simon et al., 2011). We then modelled the top 100 SNPs associated with material wealth against forehead patch size and laying date.

***Simulations of dispersal distances across the observed distribution of material wealth***

We used a simulation to investigate whether the observed relationship between natal and first breeding material wealth could be explained by short natal dispersal distances in relation to spatial autocorrelation in material wealth among all the available breeding territories. For all known recruits (individuals marked as juveniles and observed returning to breed as adults in the study area) we used the observed natal territory as a starting point for the simulation. We randomly drew a dispersal distance from the empirical distribution of dispersal distances of all the known recruits, and constructed a circle centered at the natal territory and with a radius equal to the randomly drawn distance. The material wealth of the territory closest to this circle among all territories for which we had material wealth data was used as the simulated material wealth in the first breeding attempt of the recruit. In this way, the only link between natal and simulated breeding material wealth is due to random dispersal distance and material wealth spatial layout. The simulation was repeated 500 times, each time recording the OLS estimates of the intercept and slope of breeding vs natal material wealth within each of three dispersal distance classes (0-500m, 500-1000m and over 1000m).

***Supporting discussion***

We found little evidence suggesting that the observed inheritance of material wealth reflects heritable variation in measured phenotypic fitness-related traits. There was no evidence for either phenotypic or genetic correlations between material wealth and measured morphological and behavioral traits (results reported in the main text). Since we especially expected that birds that arrive early to the breeding ground and that males with large forehead patch sizes (i.e. known to have a competitive advantage in disputes over territories Pärt & Qvarnström 1997), would be able to acquire (i.e. in this case find, assess and defend) territories with high levels of material wealth, we included these two traits in down-stream analyses. In these analyses we tested if the top 100 SNPs associated with material wealth in the GWAS analysis also were associated with these traits. However, we found no such indications. One possible explanation for these unexpected results (i.e. the lack of evidence suggesting that the observed inheritance of material wealth reflects heritable variation in measured phenotypic fitness-related traits), is that the birds lack information about the relative material wealth of all available breeding territories. Since flycatchers are migratory and highly time-constrained as they need to onset breeding shortly after arrival at the breeding grounds, then raise offspring and moult all feathers before returning to Africa, they may only manage to assess the material wealth of breeding territories in the close proximity to their breeding location (or to their parent’s breeding location as juveniles). Previous studies on collared flycatchers show that individuals base consecutive selection of breeding sites on their own past breeding experience and the reproductive success of neighbors (Doligez et al., 2002). This probably means that they may improve their material wealth across years but only on a very local scale that generally do not extend beyond the autocorrelation in material wealth among all the breeding territories, as suggested by our simulations. Previous work also show that individuals experience a fitness advantage by breeding close to their natal site (Pärt, 1991, 1994) suggesting that they lack accurate information about the material wealth of sites further away. Our analyses of natal dispersal together with the simulations, instate indicate that the inheritance of material wealth largely is explained by philopatry that causes a conservative distribution in material wealth among family lineages.

**Figure S1**: Illustration of mean caterpillar larvae biomass (i.e. estimated as caterpillar frass) produced during the nestling phase of the flycatcher breeding season in 11 common tree species in 2013–2014.


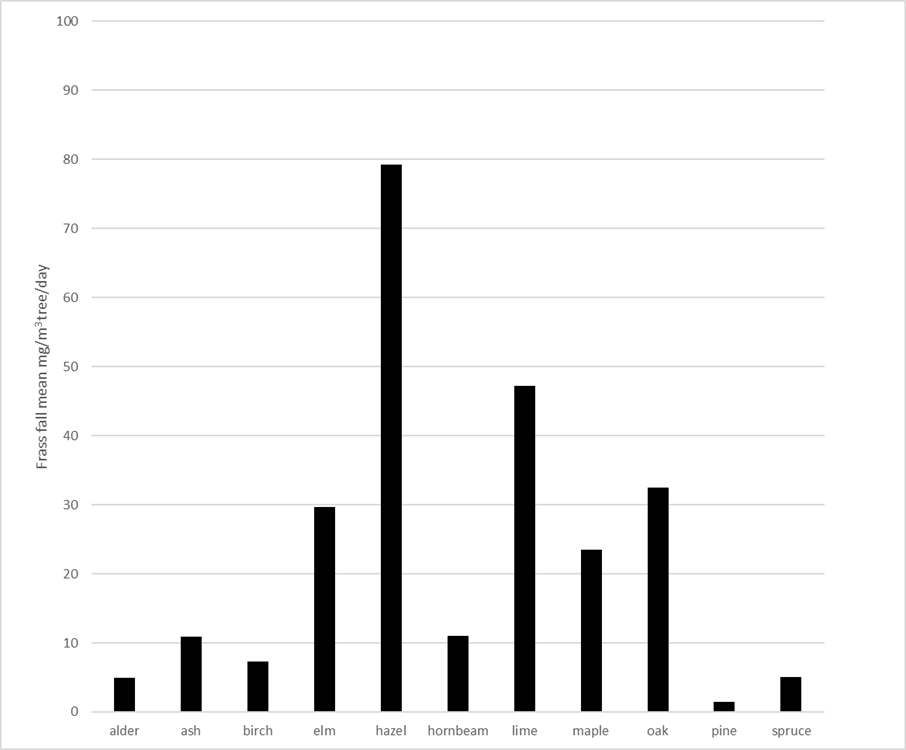


**Figure S2:** Variograms representing the amount of semivariance in material wealth at increasing distances, corresponding to the scales depicted on the map in figure 2A, i.e. two big forest patches (Horns Kungsgård and Halltorps Hage), the Northern versus Southern areas, and all areas on Öland.


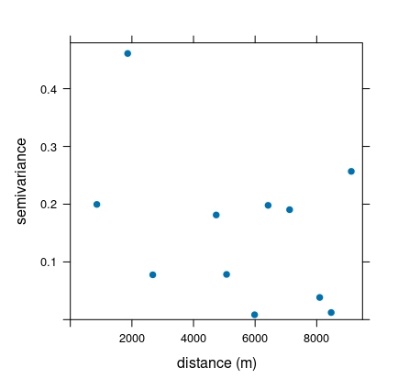


Southern areas


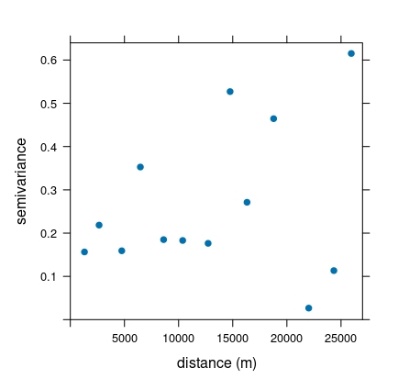


All areas on Öland


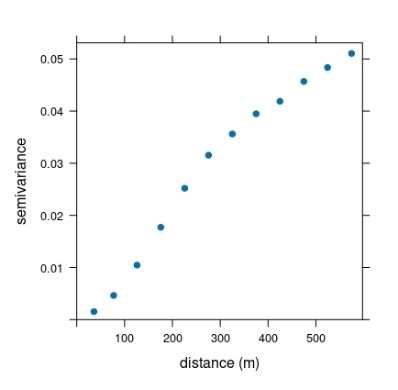


Horns Kungsgård


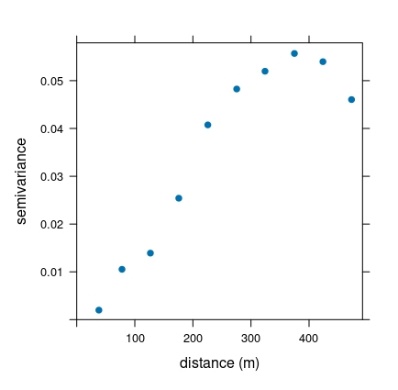


Halltorps hage


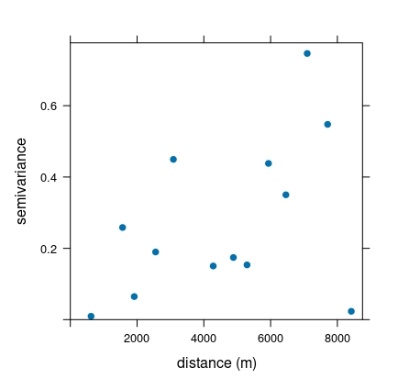


Northern areas

**Table S1:** Results of an animal model estimating the genetic heritability of wealth

| **Parameter** | **posterior mode** | **95% CI** |
| --- | --- | --- |
| h^2^ | 0.232 | 0.16 to 0.29 |
| pe^2^ | 0.264 | 0.20 to 0.34 |
| year^2^ | 0.280 | 0.18 to 0.45 |

**Table S2:** Results of a linear mixed model comparing the material wealth of the first breeding territory of returning adults that had been cross-fostered between different nests as nestlings, and the material wealth of their foster and genetic parents.

|  | **material wealth of cross-fostered recruiting indidivuals** | | | |  |  |
| --- | --- | --- | --- | --- | --- | --- |
| *fixed effects* | *estimate* | *std.error* | *t-value* | *p-value* |  |  |
| Intercept | 0.12 | 0.24 | 0.49 | 0.629 |  |  |
| Material wealth foster parents | 0.58 | 0.13 | 4.35 | **<0.001** |  |  |
| Material wealth genetic parents | 0.27 | 0.18 | 1.49 | 0.144 |  |  |
| **Random Effects** *Variance N* | | | | |  |  |
| Foster nest  Genetic nest | 0.05 44  0.00 45 | | | |  |  |
| Residuals | 0.05 | | | |  |  |

**Table S3:** Results of animal models estimating genetic correlations between material wealth and fitness related traits.

| **Trait** | **genetic correlation** | **95% CI** |
| --- | --- | --- |
| Forehead patch size | -0.021 | -0.13 to 0.10 |
| Laying date | 0.011 | -0.53 to 0.73 |
| Hatching success | 0.713 | -0.84 to 0.97 |
| Fledging success | -0.086 | -0.82 to 0.26 |
|  |  |  |

**Table S4:** List of top 100 SNPs most highly associated with wealth (i.e. the 100 lowest p-values).

| **Name** | **Chromosome** | **Position** | **P value** | **Ensembl Gene ID** | **Gene name** |
| --- | --- | --- | --- | --- | --- |
| N00160:1512798 | 20 | 1512798 | 5.49e-06 | ENSFALG00000012755 | EPB41L1 |
|  |  |  |  | ENSFALG00000025812 |  |
|  |  |  |  | ENSFALG00000012779 |  |
|  |  |  |  | ENSFALG00000027527 |  |
|  |  |  |  | ENSFALG00000023306 |  |
|  |  |  |  | ENSFALG00000026985 |  |
|  |  |  |  | ENSFALG00000028273 |  |
|  |  |  |  | ENSFALG00000025785 |  |
|  |  |  |  | ENSFALG00000026613 |  |
|  |  |  |  | ENSFALG00000018909 |  |
|  |  |  |  | ENSFALG00000010589 | AHCY |
| N00057:4273907 | 12 | 6891760 | 3.01e-05 | ENSFALG00000008880 | DNAH1 |
|  |  |  |  | ENSFALG00000008890 |  |
|  |  |  |  | ENSFALG00000008877 | BAP1 |
|  |  |  |  | ENSFALG00000008851 | SEMA3G |
|  |  |  |  | ENSFALG00000008839 | ACY1 |
|  |  |  |  | ENSFALG00000008838 | ABHD14A |
|  |  |  |  | ENSFALG00000008831 | PCBP4 |
|  |  |  |  | ENSFALG00000023354 |  |
|  |  |  |  | ENSFALG00000008829 | PARP3 |
|  |  |  |  | ENSFALG00000025494 |  |
|  |  |  |  | ENSFALG00000023225 |  |
|  |  |  |  | ENSFALG00000008828 | RBM5 |
|  |  |  |  | ENSFALG00000008826 |  |
|  |  |  |  | ENSFALG00000008821 | GNAT1 |
|  |  |  |  | ENSFALG00000008814 |  |
|  |  |  |  | ENSFALG00000008803 |  |
|  |  |  |  | ENSFALG00000023821 |  |
| N00022:4411883 | 3 | 93756290 | 3.07e-05 | ENSFALG00000025903 |  |
| N00056:3443674 | 21 | 2971560 | 6.65e-05 | ENSFALG00000009964 |  |
|  |  |  |  | ENSFALG00000023771 |  |
|  |  |  |  | ENSFALG00000025668 |  |
|  |  |  |  | ENSFALG00000026791 |  |
|  |  |  |  | ENSFALG00000009952 |  |
|  |  |  |  | ENSFALG00000009951 | LRRC38 |
|  |  |  |  | ENSFALG00000023476 |  |
|  |  |  |  | ENSFALG00000024344 |  |
|  |  |  |  | ENSFALG00000009941 |  |
| N00068:1677920 | 18 | 4188805 | 1.02e-04 | ENSFALG00000027284 |  |
|  |  |  |  | ENSFALG00000028182 |  |
| N00144:1331040 | 17 | 1331040 | 1.12e-04 | ENSFALG00000024213 |  |
|  |  |  |  | ENSFALG00000005361 |  |
|  |  |  |  | ENSFALG00000005391 |  |
|  |  |  |  | ENSFALG00000005401 | ALAD |
|  |  |  |  | ENSFALG00000005423 |  |
|  |  |  |  | ENSFALG00000023446 | BSPRY |
|  |  |  |  | ENSFALG00000005419 | WDR31 |
|  |  |  |  | ENSFALG00000024795 | SLC31A1 |
| N00101:2126115 | 9 | 2699210 | 1.37e-04 | ENSFALG00000003596 | KLHL6 |
|  |  |  |  | ENSFALG00000014547 |  |
|  |  |  |  | ENSFALG00000014529 | AMER3 |
|  |  |  |  | ENSFALG00000003578 |  |
| N00051:5526717 | 24 | 2482643 | 1.42e-04 | ENSFALG00000028552 |  |
|  |  |  |  | ENSFALG00000026097 |  |
|  |  |  |  | ENSFALG00000024285 | NECTIN1 |
|  |  |  |  | ENSFALG00000026494 |  |
| N00037:309347 | 10 | 2575138 | 1.54e-04 | ENSFALG00000009444 | HCN4 |
|  |  |  |  | ENSFALG00000025059 |  |
|  |  |  |  | ENSFALG00000028053 |  |
|  |  |  |  | ENSFALG00000009473 | NEO1 |
| N00019:8226533 | 7 | 9486036 | 2.75e-04 | ENSFALG00000027474 |  |
|  |  |  |  | ENSFALG00000025461 |  |
|  |  |  |  | ENSFALG00000004330 | SLC39A10 |
|  |  |  |  | ENSFALG00000004327 |  |
|  |  |  |  | ENSFALG00000004323 | STK17B |
| N00063:3702339 | 20 | 12270448 | 2.78e-04 | ENSFALG00000010738 | APCDD1L |
|  |  |  |  | ENSFALG00000010754 | VAPB |
|  |  |  |  | ENSFALG00000010774 |  |
|  |  |  |  | ENSFALG00000023970 | C20orf85 |
| N00025:4590981 | 11 | 6253349 | 2.82e-04 | ENSFALG00000023736 |  |
|  |  |  |  | ENSFALG00000023672 |  |
|  |  |  |  | ENSFALG00000006846 |  |
| N00139:1099103 | 10 | 1099103 | 2.84e-04 | ENSFALG00000010716 | SCAPER |
|  |  |  |  | ENSFALG00000010720 | RCN2 |
|  |  |  |  | ENSFALG00000026246 | PSTPIP1 |
|  |  |  |  | ENSFALG00000028955 | TSPAN3 |
|  |  |  |  | ENSFALG00000010742 | PEAK1 |
| N00097:3016119 | 11 | 18711048 | 3.04e-04 | ENSFALG00000007790 | SLC7A10 |
| N00017:6549607 | 4 | 15341288 | 3.47e-04 | ENSFALG00000027094 | SCD5 |
|  |  |  |  | ENSFALG00000026606 |  |
|  |  |  |  | ENSFALG00000025686 | TMEM150C |
|  |  |  |  | ENSFALG00000023501 | ENOPH1 |
|  |  |  |  | ENSFALG00000005709 | HNRNPDL |
|  |  |  |  | ENSFALG00000005716 | HNRNPD |
|  |  |  |  | ENSFALG00000005750 |  |
|  |  |  |  | ENSFALG00000005794 |  |
| N00028:2374372 | 14 | 6811347 | 3.95e-04 | ENSFALG00000028720 | LITAF |
|  |  |  |  | ENSFALG00000003935 | TXNDC11 |
|  |  |  |  | ENSFALG00000014750 | SNN |
|  |  |  |  | ENSFALG00000003931 |  |
|  |  |  |  | ENSFALG00000023147 |  |
|  |  |  |  | ENSFALG00000025731 |  |
|  |  |  |  | ENSFALG00000024104 |  |
|  |  |  |  | ENSFALG00000003919 | BFAR |
|  |  |  |  | ENSFALG00000003911 | PARN |
| N00041:2670654 | 1 | 1 | 4.27e-04 | ENSFALG00000008231 |  |
|  |  |  |  | ENSFALG00000028061 |  |
|  |  |  |  | ENSFALG00000008183 | ILK |
| N00192:178301 | 19 | 4844512 | 4.28e-04 | ENSFALG00000009380 |  |
|  |  |  |  | ENSFALG00000009409 | CUX1 |
| N00046:4578120 | 8 | 24473650 | 4.40e-04 | ENSFALG00000002708 | KIF14 |
|  |  |  |  | ENSFALG00000014467 | ZNF281 |
|  |  |  |  | ENSFALG00000028358 | NR5A2 |
| N00013:14382513 | 5 | 61945682 | 4.43e-04 | ENSFALG00000003863 | STYX |
|  |  |  |  | ENSFALG00000029088 |  |
|  |  |  |  | ENSFALG00000003847 |  |
|  |  |  |  | ENSFALG00000003873 | PSMC6 |
|  |  |  |  | ENSFALG00000003881 | ERO1A |
|  |  |  |  | ENSFALG00000024805 | GPR137C |
|  |  |  |  | ENSFALG00000026882 |  |
|  |  |  |  | ENSFALG00000003892 | TXNDC16 |
| N00196:405816 | 20 | 7782891 | 4.43e-04 | ENSFALG00000005434 | POFUT1 |
|  |  |  |  | ENSFALG00000026625 | PLAGL2 |
|  |  |  |  | ENSFALG00000005457 | TM9SF4 |
|  |  |  |  | ENSFALG00000005462 | HCK |
|  |  |  |  | ENSFALG00000022982 |  |
|  |  |  |  | ENSFALG00000028990 |  |
|  |  |  |  | ENSFALG00000024547 | XKR7 |
|  |  |  |  | ENSFALG00000005472 | PDRG1 |
|  |  |  |  | ENSFALG00000005480 | DUSP15 |
|  |  |  |  | ENSFALG00000005506 |  |
|  |  |  |  | ENSFALG00000025622 | FOXS1 |
|  |  |  |  | ENSFALG00000005519 |  |
|  |  |  |  | ENSFALG00000005528 | TPX2 |
|  |  |  |  | ENSFALG00000023035 | BCL2L1 |
|  |  |  |  | ENSFALG00000005648 | HM13 |
|  |  |  |  | ENSFALG00000024796 | REM1 |
|  |  |  |  | ENSFALG00000014163 | SOX12 |
|  |  |  |  | ENSFALG00000005680 | TBC1D20 |
| N00103:2369846 | 4 | 4278246 | 4.90e-04 | ENSFALG00000008210 |  |
|  |  |  |  | ENSFALG00000008206 | BBS12 |
|  |  |  |  | ENSFALG00000008194 | FGF2 |
|  |  |  |  | ENSFALG00000023375 | NUDT6 |
|  |  |  |  | ENSFALG00000008181 | SPATA5 |
| N00067:4037496 | 8 | 30595067 | 5.03e-04 | ENSFALG00000023726 |  |
|  |  |  |  | ENSFALG00000025173 |  |
|  |  |  |  | ENSFALG00000027153 |  |
|  |  |  |  | ENSFALG00000028654 |  |
|  |  |  |  | ENSFALG00000026687 |  |
|  |  |  |  | ENSFALG00000012778 |  |
|  |  |  |  | ENSFALG00000012808 |  |
|  |  |  |  | ENSFALG00000019896 |  |
| N00012:13849003 | 15 | 1094017 | 5.12e-04 | ENSFALG00000009481 |  |
|  |  |  |  | ENSFALG00000009466 | GLT1D1 |
|  |  |  |  | ENSFALG00000009459 | SLC15A4 |
|  |  |  |  | ENSFALG00000009443 | TMEM132C |
| N00192:890906 | 19 | 5557117 | 5.50e-04 | ENSFALG00000026877 |  |
|  |  |  |  | ENSFALG00000009669 | TMEM120A |
|  |  |  |  | ENSFALG00000009688 | POR |
|  |  |  |  | ENSFALG00000009693 |  |
|  |  |  |  | ENSFALG00000009696 |  |
|  |  |  |  | ENSFALG00000009701 | MMP28 |
|  |  |  |  | ENSFALG00000027238 |  |
|  |  |  |  | ENSFALG00000027159 | RASL10B |
|  |  |  |  | ENSFALG00000009715 | AP2B1 |
| N00039:6557951 | 17 | 11226113 | 5.54e-04 | ENSFALG00000002081 | NEK6 |
|  |  |  |  | ENSFALG00000002108 | PSMB7 |
|  |  |  |  | ENSFALG00000002127 | ADGRD2 |
|  |  |  |  | ENSFALG00000002130 | NR5A1 |
|  |  |  |  | ENSFALG00000002133 | NR6A1 |
|  |  |  |  | ENSFALG00000024786 |  |
| N00036:4565034 | 14 | 13755752 | 5.65e-04 | ENSFALG00000024004 |  |
|  |  |  |  | ENSFALG00000027671 | FOXL3 |
|  |  |  |  | ENSFALG00000013961 | FAM20C |
| N00067:1103926 | 8 | 27661497 | 5.94e-04 | ENSFALG00000012246 | PLPP3 |
|  |  |  |  | ENSFALG00000023743 |  |
|  |  |  |  | ENSFALG00000012259 | PRKAA2 |
|  |  |  |  | ENSFALG00000012284 |  |
|  |  |  |  | ENSFALG00000012292 | C8A |
|  |  |  |  | ENSFALG00000012302 | C8B |
|  |  |  |  | ENSFALG00000012314 | DAB1 |
| N00028:8047304 | 14 | 1138415 | 6.03e-04 | ENSFALG00000004730 |  |
|  |  |  |  | ENSFALG00000025144 |  |
|  |  |  |  | ENSFALG00000004697 | METTL26 |
|  |  |  |  | ENSFALG00000004693 | WFIKKN1 |
|  |  |  |  | ENSFALG00000026433 | RAB40C |
|  |  |  |  | ENSFALG00000004689 | PIGQ |
|  |  |  |  | ENSFALG00000014870 |  |
|  |  |  |  | ENSFALG00000004687 |  |
|  |  |  |  | ENSFALG00000028256 |  |
|  |  |  |  | ENSFALG00000023053 |  |
|  |  |  |  | ENSFALG00000004681 | CAPN15 |
| N00033:8050878 | 10 | 19218608 | 6.70e-04 | ENSFALG00000012785 | LACTB |
|  |  |  |  | ENSFALG00000024664 | RPL4 |
|  |  |  |  | ENSFALG00000028530 | SNORD18 |
|  |  |  |  | ENSFALG00000023838 | SNORD18 |
|  |  |  |  | ENSFALG00000015648 | SNORD16 |
|  |  |  |  | ENSFALG00000025045 | SNORD18 |
|  |  |  |  | ENSFALG00000016206 | SNORD16 |
|  |  |  |  | ENSFALG00000012802 | ZWILCH |
|  |  |  |  | ENSFALG00000012819 | LCTL |
|  |  |  |  | ENSFALG00000023761 |  |
| N00094:1116402 | 6 | 35067430 | 7.21e-04 | ENSFALG00000001201 | PTPRE |
|  |  |  |  | ENSFALG00000001204 |  |
|  |  |  |  | ENSFALG00000023643 |  |
| N00210:969837 | 27 | 2489248 | 7.50e-04 | ENSFALG00000000906 |  |
|  |  |  |  | ENSFALG00000000894 |  |
|  |  |  |  | ENSFALG00000015314 |  |
|  |  |  |  | ENSFALG00000015309 |  |
|  |  |  |  | ENSFALG00000026798 |  |
|  |  |  |  | ENSFALG00000015304 |  |
|  |  |  |  | ENSFALG00000015289 |  |
|  |  |  |  | ENSFALG00000027660 |  |
|  |  |  |  | ENSFALG00000000887 |  |
|  |  |  |  | ENSFALG00000015275 |  |
|  |  |  |  | ENSFALG00000000713 |  |
|  |  |  |  | ENSFALG00000000865 |  |
|  |  |  |  | ENSFALG00000024199 |  |
|  |  |  |  | ENSFALG00000000842 |  |
|  |  |  |  | ENSFALG00000000854 |  |
|  |  |  |  | ENSFALG00000027972 |  |
|  |  |  |  | ENSFALG00000000834 | MPP3 |
| N00068:3685645 | 18 | 6196530 | 7.68e-04 | ENSFALG00000001251 | CDR2L |
|  |  |  |  | ENSFALG00000001252 | HID1 |
|  |  |  |  | ENSFALG00000001254 | OTOP3 |
|  |  |  |  | ENSFALG00000001257 | OTOP2 |
|  |  |  |  | ENSFALG00000001261 | USH1G |
|  |  |  |  | ENSFALG00000001263 | FADS6 |
|  |  |  |  | ENSFALG00000001271 |  |
|  |  |  |  | ENSFALG00000001279 | GRIN2C |
|  |  |  |  | ENSFALG00000001282 | TMEM104 |
|  |  |  |  | ENSFALG00000001288 | NAT9 |
|  |  |  |  | ENSFALG00000026664 | NHERF1 |
| N00213:55234 | 7 | 942728 | 8.07e-04 | ENSFALG00000003836 | ASB1 |
|  |  |  |  | ENSFALG00000003831 | TRAF3IP1 |
|  |  |  |  | ENSFALG00000003826 |  |
|  |  |  |  | ENSFALG00000000620 |  |
| N00044:775308 | 6 | 31941740 | 8.55e-04 | ENSFALG00000026547 | PLPP4 |
|  |  |  |  | ENSFALG00000024601 |  |
|  |  |  |  | ENSFALG00000002154 | WDR11 |
| N00040:1031698 | 23 | 6370750 | 8.62e-04 | ENSFALG00000000543 | PABPC4 |
|  |  |  |  | ENSFALG00000000542 | HEYL |
|  |  |  |  | ENSFALG00000000539 | NT5C1A |
|  |  |  |  | ENSFALG00000000538 | HPCAL4 |
|  |  |  |  | ENSFALG00000000537 |  |
|  |  |  |  | ENSFALG00000028578 |  |
|  |  |  |  | ENSFALG00000027711 |  |
|  |  |  |  | ENSFALG00000000526 | MYCL |
|  |  |  |  | ENSFALG00000000524 | MFSD2A |
|  |  |  |  | ENSFALG00000000523 | CAP1 |
|  |  |  |  | ENSFALG00000000522 | PPT1 |
|  |  |  |  | ENSFALG00000000520 | RPL11 |
|  |  |  |  | ENSFALG00000000519 |  |
|  |  |  |  | ENSFALG00000000518 | PITHD1 |
|  |  |  |  | ENSFALG00000028382 | LYPLA2 |
|  |  |  |  | ENSFALG00000000514 | GALE |
|  |  |  |  | ENSFALG00000000512 | HMGCL |
|  |  |  |  | ENSFALG00000000508 | FUCA1 |
|  |  |  |  | ENSFALG00000014254 | CNR2 |
| N00115:204780 | 5 | 64519815 | 8.75e-04 | ENSFALG00000001660 | LRFN5 |
| N00028:1508104 | 14 | 7677615 | 9.03e-04 | ENSFALG00000003895 |  |
|  |  |  |  | ENSFALG00000003888 | SNX29 |
|  |  |  |  | ENSFALG00000003880 | GSPT1 |
| N00135:1097583 | 6 | 1097583 | 1.04e-03 | ENSFALG00000012946 | HERC4 |
|  |  |  |  | ENSFALG00000025663 |  |
|  |  |  |  | ENSFALG00000012952 | MYPN |
|  |  |  |  | ENSFALG00000014082 | ATOH7 |
|  |  |  |  | ENSFALG00000012957 | RUFY2 |
|  |  |  |  | ENSFALG00000012964 | HNRNPH3 |
|  |  |  |  | ENSFALG00000012984 | DNA2 |
|  |  |  |  | ENSFALG00000013006 | TET1 |
|  |  |  |  | ENSFALG00000027117 | SLC25A16 |
| N00512:22691 | 6 | 2070317 | 1.07e-03 | ENSFALG00000013275 | CDH23 |
|  |  |  |  | ENSFALG00000013374 | SLC29A3 |
|  |  |  |  | ENSFALG00000013380 | UNC5B |
| N00210:553365 | 27 | 2905720 | 1.10e-03 | ENSFALG00000000786 | RND2 |
|  |  |  |  | ENSFALG00000025296 | VAT1 |
|  |  |  |  | ENSFALG00000000781 |  |
|  |  |  |  | ENSFALG00000000772 | RPL27 |
|  |  |  |  | ENSFALG00000000768 | RUNDC1 |
|  |  |  |  | ENSFALG00000024118 | PTGES3L |
|  |  |  |  | ENSFALG00000000757 | AARSD1 |
|  |  |  |  | ENSFALG00000000748 |  |
|  |  |  |  | ENSFALG00000000739 | PSME3 |
|  |  |  |  | ENSFALG00000000729 |  |
|  |  |  |  | ENSFALG00000025771 | CNTD1 |
|  |  |  |  | ENSFALG00000000723 | WNK4 |
|  |  |  |  | ENSFALG00000028719 | VPS25 |
|  |  |  |  | ENSFALG00000000719 | RAMP2 |
|  |  |  |  | ENSFALG00000000715 | EZH1 |
|  |  |  |  | ENSFALG00000000712 | CNTNAP1 |
|  |  |  |  | ENSFALG00000027084 | CCR10 |
|  |  |  |  | ENSFALG00000000709 | PLEKHH3 |
|  |  |  |  | ENSFALG00000000706 | TUBG1 |
|  |  |  |  | ENSFALG00000023962 |  |
|  |  |  |  | ENSFALG00000023055 | PSMC3IP |
|  |  |  |  | ENSFALG00000000692 | MLX |
|  |  |  |  | ENSFALG00000000689 | COASY |
|  |  |  |  | ENSFALG00000025595 | NAGLU |
|  |  |  |  | ENSFALG00000000686 | HSD17B1 |
|  |  |  |  | ENSFALG00000000677 | ATP6V0A1 |
| N00100:2906555 | 13 | 13377543 | 1.14e-03 | ENSFALG00000009707 | TENM2 |
| N00003:18580075 | 2 | 25743539 | 1.18e-03 | ENSFALG00000010302 | PPP1R9A |
|  |  |  |  | ENSFALG00000025037 |  |
|  |  |  |  | ENSFALG00000010284 | ASB4 |
|  |  |  |  | ENSFALG00000010276 | PDK4 |
|  |  |  |  | ENSFALG00000010272 | DYNC1I1 |
| N00039:2494836 | 17 | 7162998 | 1.22e-03 | ENSFALG00000001447 | ABL1 |
|  |  |  |  | ENSFALG00000025929 |  |
|  |  |  |  | ENSFALG00000001448 | FIBCD1 |
|  |  |  |  | ENSFALG00000001449 | LAMC3 |
|  |  |  |  | ENSFALG00000027282 | AIF1L |
|  |  |  |  | ENSFALG00000028450 |  |
|  |  |  |  | ENSFALG00000001451 | NUP214 |
| N00144:1703476 | 17 | 1703476 | 1.25e-03 | ENSFALG00000005521 | PNPLA7 |
|  |  |  |  | ENSFALG00000005579 | NSMF |
|  |  |  |  | ENSFALG00000024714 |  |
|  |  |  |  | ENSFALG00000028325 |  |
|  |  |  |  | ENSFALG00000005595 |  |
|  |  |  |  | ENSFALG00000005604 |  |
| N00036:5557332 | 14 | 14748050 | 1.30e-03 | ENSFALG00000013970 | LMTK2 |
|  |  |  |  | ENSFALG00000013971 |  |
|  |  |  |  | ENSFALG00000013972 |  |
|  |  |  |  | ENSFALG00000013973 |  |
|  |  |  |  | ENSFALG00000013974 |  |
|  |  |  |  | ENSFALG00000013975 |  |
|  |  |  |  | ENSFALG00000013976 | PMS2 |
|  |  |  |  | ENSFALG00000023978 | AIMP2 |
|  |  |  |  | ENSFALG00000013978 | EIF2AK1 |
|  |  |  |  | ENSFALG00000013980 |  |
|  |  |  |  | ENSFALG00000013981 | CYTH3 |
| N00072:1083096 | 18 | 8484251 | 1.31e-03 | ENSFALG00000002277 | TRIM25 |
|  |  |  |  | ENSFALG00000002278 | COIL |
|  |  |  |  | ENSFALG00000002280 | SCPEP1 |
|  |  |  |  | ENSFALG00000002281 | RAB11FIP4 |
|  |  |  |  | ENSFALG00000016133 |  |
|  |  |  |  | ENSFALG00000016035 |  |
| N00290:20636 | 8 | 31091652 | 1.37e-03 | ENSFALG00000012942 | WLS |
|  |  |  |  | ENSFALG00000012976 |  |
|  |  |  |  | ENSFALG00000012996 | RPE65 |
|  |  |  |  | ENSFALG00000013009 | DEPDC1 |
|  |  |  |  | ENSFALG00000013034 |  |
|  |  |  |  | ENSFALG00000028454 | ANKRD13C |
|  |  |  |  | ENSFALG00000001217 | SRSF11 |
|  |  |  |  | ENSFALG00000001226 | LRRC40 |
|  |  |  |  | ENSFALG00000001237 | LRRC7 |
| N00014:11900433 | 2 | 1 | 1.43e-03 | ENSFALG00000025394 |  |
|  |  |  |  | ENSFALG00000013203 | CHPF2 |
|  |  |  |  | ENSFALG00000013201 | ABCF2 |
| N00012:4939100 | 15 | 10003920 | 1.44e-03 | ENSFALG00000028169 |  |
|  |  |  |  | ENSFALG00000024854 |  |
|  |  |  |  | ENSFALG00000007792 | TBX3 |
|  |  |  |  | ENSFALG00000026464 |  |
| N00251:90732 | 24 | 824348 | 1.48e-03 | ENSFALG00000023395 |  |
|  |  |  |  | ENSFALG00000022872 |  |
|  |  |  |  | ENSFALG00000005527 | BUD13 |
|  |  |  |  | ENSFALG00000005541 | ZPR1 |
|  |  |  |  | ENSFALG00000027449 | APOA5 |
|  |  |  |  | ENSFALG00000005578 | APOA4 |
|  |  |  |  | ENSFALG00000005591 |  |
|  |  |  |  | ENSFALG00000005608 | SIK3 |
| N00206:505439 | 3 | 42947407 | 1.49e-03 | ENSFALG00000003307 | RHOU |
|  |  |  |  | ENSFALG00000023834 | EXOC8 |
|  |  |  |  | ENSFALG00000027582 | SPRTN |
|  |  |  |  | ENSFALG00000028954 |  |
|  |  |  |  | ENSFALG00000027867 |  |
|  |  |  |  | ENSFALG00000003260 | TSNAX |
| N00283:14780 | 8 | 31553931 | 1.50e-03 | ENSFALG00000001249 | NEGR1 |
|  |  |  |  | ENSFALG00000026959 |  |
|  |  |  |  | ENSFALG00000023525 |  |
| N00089:2089800 | 1 | 94271774 | 1.53e-03 | ENSFALG00000005249 |  |
|  |  |  |  | ENSFALG00000005269 | POLQ |
|  |  |  |  | ENSFALG00000005282 | STXBP5L |
| N00013:12586010 | 5 | 60149179 | 1.56e-03 | ENSFALG00000003614 | SLC35F4 |
|  |  |  |  | ENSFALG00000003624 | CCDC198 |
|  |  |  |  | ENSFALG00000003631 | NAA30 |
|  |  |  |  | ENSFALG00000003651 | AP5M1 |
|  |  |  |  | ENSFALG00000003680 | EXOC5 |
| N00068:2924799 | 18 | 5435684 | 1.61e-03 | ENSFALG00000000982 | SRP68 |
|  |  |  |  | ENSFALG00000000987 |  |
|  |  |  |  | ENSFALG00000025835 |  |
|  |  |  |  | ENSFALG00000000992 | CDK3 |
|  |  |  |  | ENSFALG00000024866 |  |
|  |  |  |  | ENSFALG00000001005 | MRPL38 |
|  |  |  |  | ENSFALG00000028723 | TRIM65 |
|  |  |  |  | ENSFALG00000001020 |  |
|  |  |  |  | ENSFALG00000026954 | WBP2 |
|  |  |  |  | ENSFALG00000024919 |  |
|  |  |  |  | ENSFALG00000001027 | UNC13D |
|  |  |  |  | ENSFALG00000001035 | UNK |
|  |  |  |  | ENSFALG00000001052 |  |
|  |  |  |  | ENSFALG00000028436 |  |
|  |  |  |  | ENSFALG00000001061 | GALK1 |
|  |  |  |  | ENSFALG00000001064 | ITGB4 |
| N00120:185519 | 2 | 3676332 | 1.64e-03 | ENSFALG00000000828 | GHRHR |
|  |  |  |  | ENSFALG00000000812 | AQP1 |
|  |  |  |  | ENSFALG00000027755 |  |
|  |  |  |  | ENSFALG00000000808 | MINDY4 |
| N00052:1527335 | 19 | 10406338 | 1.64e-03 | ENSFALG00000005291 | LHX1 |
|  |  |  |  | ENSFALG00000005270 | AATF |
|  |  |  |  | ENSFALG00000005248 | ACACA |
|  |  |  |  | ENSFALG00000005233 | TADA2A |
| N00289:411609 | 27 | 2131366 | 1.66e-03 | ENSFALG00000027649 |  |
|  |  |  |  | ENSFALG00000026894 |  |
|  |  |  |  | ENSFALG00000024430 |  |
|  |  |  |  | ENSFALG00000028417 |  |
|  |  |  |  | ENSFALG00000024605 |  |
|  |  |  |  | ENSFALG00000027890 |  |
|  |  |  |  | ENSFALG00000026152 |  |
|  |  |  |  | ENSFALG00000026546 |  |
|  |  |  |  | ENSFALG00000028511 |  |
|  |  |  |  | ENSFALG00000014101 |  |
|  |  |  |  | ENSFALG00000028451 |  |
|  |  |  |  | ENSFALG00000023530 |  |
|  |  |  |  | ENSFALG00000027869 |  |
| N00036:6937702 | 14 | 16128420 | 1.71e-03 | ENSFALG00000014051 |  |
| N00004:1159042 | 2 | 5025892 | 1.76e-03 | ENSFALG00000006624 | OXSR1 |
|  |  |  |  | ENSFALG00000028804 |  |
|  |  |  |  | ENSFALG00000025666 |  |
| N00166:261025 | 9 | 25547130 | 1.83e-03 | ENSFALG00000004742 | VEPH1 |
|  |  |  |  | ENSFALG00000004760 | CCNL1 |
|  |  |  |  | ENSFALG00000004778 | LEKR1 |
|  |  |  |  | ENSFALG00000027016 |  |
|  |  |  |  | ENSFALG00000004781 | TIPARP |
|  |  |  |  | ENSFALG00000004786 | SSR3 |
|  |  |  |  | ENSFALG00000004804 | KCNAB1 |
| N00037:2736868 | 10 | 5002659 | 1.88e-03 | ENSFALG00000023776 |  |
|  |  |  |  | ENSFALG00000010188 | PCSK6 |
|  |  |  |  | ENSFALG00000010195 | SNRPA1 |
|  |  |  |  | ENSFALG00000010210 |  |
|  |  |  |  | ENSFALG00000010213 | CHSY1 |
|  |  |  |  | ENSFALG00000010215 | LRRK1 |
| N00105:1411548 | 3 | 5687794 | 2.01e-03 | ENSFALG00000010942 | SPTLC3 |
|  |  |  |  | ENSFALG00000010932 | ISM1 |
|  |  |  |  | ENSFALG00000024889 | TASP1 |
| N00226:659717 | 27 | 4123801 | 2.07e-03 | ENSFALG00000025705 |  |
|  |  |  |  | ENSFALG00000000553 | CDK12 |
|  |  |  |  | ENSFALG00000000555 | MED1 |
|  |  |  |  | ENSFALG00000000557 | FBXL20 |
|  |  |  |  | ENSFALG00000000558 |  |
|  |  |  |  | ENSFALG00000025142 |  |
|  |  |  |  | ENSFALG00000019590 |  |
|  |  |  |  | ENSFALG00000028803 |  |

**Table S5**: Functional profile of the gene sets associated with the genes linked to the 100 SNPs most highly associated with material wealth in the GWAS.

| **GO ID** | **Count** | **Ontology** | **Description** |
| --- | --- | --- | --- |
| <GO:0071704> | 164 | Biological process | organic substance metabolic process |
| <GO:0050789> | 163 | Biological process | regulation of biological process |
| <GO:0044238> | 160 | Biological process | primary metabolic process |
| <GO:0050794> | 152 | Biological process | regulation of cellular process |
| <GO:0006807> | 148 | Biological process | nitrogen compound metabolic process |
| <GO:0044237> | 135 | Biological process | cellular metabolic process |
| <GO:0051716> | 98 | Biological process | cellular response to stimulus |
| <GO:0007154> | 92 | Biological process | cell communication |
| <GO:0048856> | 85 | Biological process | anatomical structure development |
| <GO:0019222> | 83 | Biological process | regulation of metabolic process |
| <GO:0007165> | 82 | Biological process | signal transduction |
| <GO:0071840> | 82 | Biological process | cellular component organization or biogenesis |
| <GO:0051234> | 78 | Biological process | establishment of localization |
| <GO:0048519> | 76 | Biological process | negative regulation of biological process |
| <GO:0048518> | 71 | Biological process | positive regulation of biological process |
| <GO:0007275> | 70 | Biological process | multicellular organism development |
| <GO:0009058> | 69 | Biological process | biosynthetic process |
| <GO:0048523> | 66 | Biological process | negative regulation of cellular process |
| <GO:0048522> | 62 | Biological process | positive regulation of cellular process |
| <GO:0048869> | 53 | Biological process | cellular developmental process |
| <GO:0048583> | 52 | Biological process | regulation of response to stimulus |
| <GO:0051641> | 48 | Biological process | cellular localization |
| <GO:0009056> | 46 | Biological process | catabolic process |
| <GO:0009653> | 46 | Biological process | anatomical structure morphogenesis |
| <GO:0009893> | 43 | Biological process | positive regulation of metabolic process |
| <GO:0023051> | 42 | Biological process | regulation of signaling |
| <GO:0033036> | 40 | Biological process | macromolecule localization |
| <GO:0042221> | 38 | Biological process | response to chemical |
| <GO:0006950> | 37 | Biological process | response to stress |
| <GO:0065008> | 37 | Biological process | regulation of biological quality |
| <GO:0009892> | 36 | Biological process | negative regulation of metabolic process |
| <GO:0051239> | 35 | Biological process | regulation of multicellular organismal process |
| <GO:0050793> | 34 | Biological process | regulation of developmental process |
| <GO:0009605> | 33 | Biological process | response to external stimulus |
| <GO:0032879> | 30 | Biological process | regulation of localization |
| <GO:0065009> | 30 | Biological process | regulation of molecular function |
| <GO:0044281> | 28 | Biological process | small molecule metabolic process |
| <GO:0007049> | 28 | Biological process | cell cycle |
| <GO:0055085> | 27 | Biological process | transmembrane transport |
| <GO:0003008> | 26 | Biological process | system process |
| <GO:0008219> | 25 | Biological process | cell death |
| <GO:0048585> | 24 | Biological process | negative regulation of response to stimulus |
| <GO:0048584> | 22 | Biological process | positive regulation of response to stimulus |
| <GO:0007267> | 21 | Biological process | cell-cell signaling |
| <GO:0009719> | 20 | Biological process | response to endogenous stimulus |
| <GO:0002682> | 19 | Biological process | regulation of immune system process |
| <GO:0048878> | 19 | Biological process | chemical homeostasis |
| <GO:0023057> | 18 | Biological process | negative regulation of signaling |
| <GO:0048646> | 18 | Biological process | anatomical structure formation involved in morphogenesis |
| <GO:0007155> | 17 | Biological process | cell adhesion |
| <GO:0023056> | 17 | Biological process | positive regulation of signaling |
| <GO:0022414> | 16 | Biological process | reproductive process |
| <GO:0006955> | 16 | Biological process | immune response |
| <GO:0022402> | 16 | Biological process | cell cycle process |
| <GO:0048870> | 16 | Biological process | cell motility |
| <GO:0008283> | 15 | Biological process | cell population proliferation |
| <GO:0051094> | 15 | Biological process | positive regulation of developmental process |
| <GO:0003006> | 14 | Biological process | developmental process involved in reproduction |
| <GO:0048871> | 14 | Biological process | multicellular organismal-level homeostasis |
| <GO:0019725> | 14 | Biological process | cellular homeostasis |
| <GO:0051707> | 14 | Biological process | response to other organism |
| <GO:0009607> | 14 | Biological process | response to biotic stimulus |
| <GO:0002684> | 13 | Biological process | positive regulation of immune system process |
| <GO:0007017> | 13 | Biological process | microtubule-based process |
| <GO:0051240> | 13 | Biological process | positive regulation of multicellular organismal process |
| <GO:0051301> | 12 | Biological process | cell division |
| <GO:0051093> | 12 | Biological process | negative regulation of developmental process |
| <GO:0030029> | 11 | Biological process | actin filament-based process |
| <GO:0140352> | 11 | Biological process | export from cell |
| <GO:0048589> | 11 | Biological process | developmental growth |
| <GO:0040008> | 11 | Biological process | regulation of growth |
| <GO:0032504> | 10 | Biological process | multicellular organism reproduction |
| <GO:0045321> | 10 | Biological process | leukocyte activation |
| <GO:0001775> | 10 | Biological process | cell activation |
| <GO:0048609> | 10 | Biological process | multicellular organismal reproductive process |
| <GO:0051241> | 10 | Biological process | negative regulation of multicellular organismal process |
| <GO:0019953> | 9 | Biological process | sexual reproduction |
| <GO:0002252> | 9 | Biological process | immune effector process |
| <GO:0040012> | 9 | Biological process | regulation of locomotion |
| <GO:0051050> | 9 | Biological process | positive regulation of transport |
| <GO:0009628> | 9 | Biological process | response to abiotic stimulus |
| <GO:0051606> | 9 | Biological process | detection of stimulus |
| <GO:0002253> | 8 | Biological process | activation of immune response |
| <GO:0042445> | 8 | Biological process | hormone metabolic process |
| <GO:0007610> | 8 | Biological process | behavior |
| <GO:0042330> | 8 | Biological process | taxis |
| <GO:0051640> | 8 | Biological process | organelle localization |
| <GO:0007389> | 7 | Biological process | pattern specification process |
| <GO:0051051> | 7 | Biological process | negative regulation of transport |
| <GO:0002440> | 6 | Biological process | production of molecular mediator of immune response |
| <GO:0032259> | 6 | Biological process | methylation |
| <GO:0016049> | 6 | Biological process | cell growth |
| <GO:0061919> | 6 | Biological process | process utilizing autophagic mechanism |
| <GO:0021700> | 6 | Biological process | developmental maturation |
| <GO:0002520> | 5 | Biological process | immune system development |
| <GO:0022412> | 5 | Biological process | cellular process involved in reproduction in multicellular organism |
| <GO:0051651> | 5 | Biological process | maintenance of location in cell |
| <GO:0001503> | 5 | Biological process | ossification |
| <GO:0009791> | 5 | Biological process | post-embryonic development |
| <GO:0035264> | 5 | Biological process | multicellular organism growth |
| <GO:0045927> | 5 | Biological process | positive regulation of growth |
| <GO:0040017> | 5 | Biological process | positive regulation of locomotion |
| <GO:0051235> | 5 | Biological process | maintenance of location |
| <GO:0002200> | 4 | Biological process | somatic diversification of immune receptors |
| <GO:0050900> | 4 | Biological process | leukocyte migration |
| <GO:0001906> | 4 | Biological process | cell killing |
| <GO:0007272> | 4 | Biological process | ensheathment of neurons |
| <GO:0071827> | 4 | Biological process | plasma lipoprotein particle organization |
| <GO:0007623> | 4 | Biological process | circadian rhythm |
| <GO:0097006> | 4 | Biological process | regulation of plasma lipoprotein particle levels |
| <GO:0002683> | 3 | Biological process | negative regulation of immune system process |
| <GO:0007163> | 3 | Biological process | establishment or maintenance of cell polarity |
| <GO:0019058> | 3 | Biological process | viral life cycle |
| <GO:0009566> | 3 | Biological process | fertilization |
| <GO:2000241> | 3 | Biological process | regulation of reproductive process |
| <GO:0001763> | 3 | Biological process | morphogenesis of a branching structure |
| <GO:0001816> | 3 | Biological process | cytokine production |
| <GO:0044706> | 3 | Biological process | multi-multicellular organism process |
| <GO:0045494> | 3 | Biological process | photoreceptor cell maintenance |
| <GO:0090130> | 3 | Biological process | tissue migration |
| <GO:0040013> | 3 | Biological process | negative regulation of locomotion |
| <GO:0031640> | 3 | Biological process | killing of cells of another organism |
| <GO:0044403> | 3 | Biological process | biological process involved in symbiotic interaction |
| <GO:0042752> | 3 | Biological process | regulation of circadian rhythm |
| <GO:0006885> | 3 | Biological process | regulation of pH |
| <GO:0001776> | 2 | Biological process | leukocyte homeostasis |
| <GO:0002262> | 2 | Biological process | myeloid cell homeostasis |
| <GO:0042440> | 2 | Biological process | pigment metabolic process |
| <GO:0006457> | 2 | Biological process | protein folding |
| <GO:0006903> | 2 | Biological process | vesicle targeting |
| <GO:0019068> | 2 | Biological process | virion assembly |
| <GO:0050792> | 2 | Biological process | regulation of viral process |
| <GO:0007566> | 2 | Biological process | embryo implantation |
| <GO:0022602> | 2 | Biological process | ovulation cycle process |
| <GO:0044703> | 2 | Biological process | multi-organism reproductive process |
| <GO:0051321> | 2 | Biological process | meiotic cell cycle |
| <GO:1903046> | 2 | Biological process | meiotic cell cycle process |
| <GO:2000242> | 2 | Biological process | negative regulation of reproductive process |
| <GO:0007586> | 2 | Biological process | digestion |
| <GO:0008340> | 2 | Biological process | determination of adult lifespan |
| <GO:0048771> | 2 | Biological process | tissue remodeling |
| <GO:0048532> | 2 | Biological process | anatomical structure arrangement |
| <GO:0045926> | 2 | Biological process | negative regulation of growth |
| <GO:0042698> | 2 | Biological process | ovulation cycle |
| <GO:0031503> | 2 | Biological process | protein-containing complex localization |
| <GO:0032963> | 1 | Biological process | collagen metabolic process |
| <GO:0070085> | 1 | Biological process | glycosylation |
| <GO:0070988> | 1 | Biological process | demethylation |
| <GO:0006949> | 1 | Biological process | syncytium formation |
| <GO:0044068> | 1 | Biological process | modulation by symbiont of host cellular process |
| <GO:0045103> | 1 | Biological process | intermediate filament-based process |
| <GO:0097194> | 1 | Biological process | execution phase of apoptosis |
| <GO:0140253> | 1 | Biological process | cell-cell fusion |
| <GO:1990748> | 1 | Biological process | cellular detoxification |
| <GO:0019048> | 1 | Biological process | modulation by virus of host process |
| <GO:0019076> | 1 | Biological process | viral release from host cell |
| <GO:0019079> | 1 | Biological process | viral genome replication |
| <GO:0044794> | 1 | Biological process | positive regulation by host of viral process |
| <GO:0048524> | 1 | Biological process | positive regulation of viral process |
| <GO:0048525> | 1 | Biological process | negative regulation of viral process |
| <GO:0061450> | 1 | Biological process | trophoblast cell migration |
| <GO:0097722> | 1 | Biological process | sperm motility |
| <GO:2000243> | 1 | Biological process | positive regulation of reproductive process |
| <GO:0002021> | 1 | Biological process | response to dietary excess |
| <GO:0019827> | 1 | Biological process | stem cell population maintenance |
| <GO:0022404> | 1 | Biological process | molting cycle process |
| <GO:0032898> | 1 | Biological process | neurotrophin production |
| <GO:0032941> | 1 | Biological process | secretion by tissue |
| <GO:0033555> | 1 | Biological process | multicellular organismal response to stress |
| <GO:0035265> | 1 | Biological process | organ growth |
| <GO:0042303> | 1 | Biological process | molting cycle |
| <GO:0045056> | 1 | Biological process | transcytosis |
| <GO:0071684> | 1 | Biological process | organism emergence from protective structure |
| <GO:0031128> | 1 | Biological process | developmental induction |
| <GO:0098727> | 1 | Biological process | maintenance of cell number |
| <GO:0033058> | 1 | Biological process | directional locomotion |
| <GO:0035821> | 1 | Biological process | modulation of process of another organism |
| <GO:1900272> | 1 | Biological process | negative regulation of long-term synaptic potentiation |
| <GO:0043903> | 1 | Biological process | regulation of biological process involved in symbiotic interaction |
| <GO:0098900> | 1 | Biological process | regulation of action potential |
| <GO:0014823> | 1 | Biological process | response to activity |
| <GO:0005622> | 197 | Cellular component | intracellular anatomical structure |
| <GO:0043226> | 178 | Cellular component | organelle |
| <GO:0005737> | 134 | Cellular component | cytoplasm |
| <GO:0016020> | 119 | Cellular component | membrane |
| <GO:0031974> | 69 | Cellular component | membrane-enclosed lumen |
| <GO:0012505> | 58 | Cellular component | endomembrane system |
| <GO:0071944> | 56 | Cellular component | cell periphery |
| <GO:0005654> | 50 | Cellular component | nucleoplasm |
| <GO:0005829> | 48 | Cellular component | cytosol |
| <GO:0099080> | 27 | Cellular component | supramolecular complex |
| <GO:0030054> | 23 | Cellular component | cell junction |
| <GO:1902494> | 21 | Cellular component | catalytic complex |
| <GO:0042995> | 21 | Cellular component | cell projection |
| <GO:0031984> | 18 | Cellular component | organelle subcompartment |
| <GO:0005576> | 16 | Cellular component | extracellular region |
| <GO:0016604> | 16 | Cellular component | nuclear body |
| <GO:0098796> | 15 | Cellular component | membrane protein complex |
| <GO:1990904> | 12 | Cellular component | ribonucleoprotein complex |
| <GO:0140535> | 11 | Cellular component | intracellular protein-containing complex |
| <GO:0140513> | 10 | Cellular component | nuclear protein-containing complex |
| <GO:0031975> | 10 | Cellular component | envelope |
| <GO:0005615> | 9 | Cellular component | extracellular space |
| <GO:0005815> | 8 | Cellular component | microtubule organizing center |
| <GO:0009986> | 8 | Cellular component | cell surface |
| <GO:0036477> | 6 | Cellular component | somatodendritic compartment |
| <GO:0044297> | 6 | Cellular component | cell body |
| <GO:0048471> | 6 | Cellular component | perinuclear region of cytoplasm |
| <GO:0098687> | 6 | Cellular component | chromosomal region |
| <GO:0005667> | 5 | Cellular component | transcription regulator complex |
| <GO:0098552> | 5 | Cellular component | side of membrane |
| <GO:0098793> | 5 | Cellular component | presynapse |
| <GO:1990351> | 4 | Cellular component | transporter complex |
| <GO:0000785> | 4 | Cellular component | chromatin |
| <GO:0030312> | 4 | Cellular component | external encapsulating structure |
| <GO:0031252> | 4 | Cellular component | cell leading edge |
| <GO:0045177> | 4 | Cellular component | apical part of cell |
| <GO:0005875> | 3 | Cellular component | microtubule associated complex |
| <GO:0032993> | 3 | Cellular component | protein-DNA complex |
| <GO:0043235> | 3 | Cellular component | receptor complex |
| <GO:0140534> | 3 | Cellular component | endoplasmic reticulum protein-containing complex |
| <GO:0000922> | 3 | Cellular component | spindle pole |
| <GO:0001650> | 3 | Cellular component | fibrillar center |
| <GO:0001917> | 3 | Cellular component | photoreceptor inner segment |
| <GO:0035869> | 3 | Cellular component | ciliary transition zone |
| <GO:0045178> | 3 | Cellular component | basal part of cell |
| <GO:0098794> | 3 | Cellular component | postsynapse |
| <GO:0098862> | 3 | Cellular component | cluster of actin-based cell projections |
| <GO:0022624> | 2 | Cellular component | proteasome accessory complex |
| <GO:0032994> | 2 | Cellular component | protein-lipid complex |
| <GO:0044815> | 2 | Cellular component | DNA packaging complex |
| <GO:0098798> | 2 | Cellular component | mitochondrial protein-containing complex |
| <GO:0030427> | 2 | Cellular component | site of polarized growth |
| <GO:0030496> | 2 | Cellular component | midbody |
| <GO:0032426> | 2 | Cellular component | stereocilium tip |
| <GO:0043204> | 2 | Cellular component | perikaryon |
| <GO:0045171> | 2 | Cellular component | intercellular bridge |
| <GO:0097546> | 2 | Cellular component | ciliary base |
| <GO:0150034> | 2 | Cellular component | distal axon |
| <GO:0000930> | 1 | Cellular component | gamma-tubulin complex |
| <GO:0000939> | 1 | Cellular component | inner kinetochore |
| <GO:0000940> | 1 | Cellular component | outer kinetochore |
| <GO:0008537> | 1 | Cellular component | proteasome activator complex |
| <GO:0016272> | 1 | Cellular component | prefoldin complex |
| <GO:0018444> | 1 | Cellular component | translation release factor complex |
| <GO:0030990> | 1 | Cellular component | intraciliary transport particle |
| <GO:0030992> | 1 | Cellular component | intraciliary transport particle B |
| <GO:0031262> | 1 | Cellular component | Ndc80 complex |
| <GO:0032300> | 1 | Cellular component | mismatch repair complex |
| <GO:0035658> | 1 | Cellular component | Mon1-Ccz1 complex |
| <GO:0036452> | 1 | Cellular component | ESCRT complex |
| <GO:0097136> | 1 | Cellular component | Bcl-2 family protein complex |
| <GO:0098636> | 1 | Cellular component | protein complex involved in cell adhesion |
| <GO:0099023> | 1 | Cellular component | vesicle tethering complex |
| <GO:0106002> | 1 | Cellular component | mCRD-mediated mRNA stability complex |
| <GO:1990062> | 1 | Cellular component | RPAP3/R2TP/prefoldin-like complex |
| <GO:1990423> | 1 | Cellular component | RZZ complex |
| <GO:0000242> | 1 | Cellular component | pericentriolar material |
| <GO:0001750> | 1 | Cellular component | photoreceptor outer segment |
| <GO:0005930> | 1 | Cellular component | axoneme |
| <GO:0009295> | 1 | Cellular component | nucleoid |
| <GO:0016363> | 1 | Cellular component | nuclear matrix |
| <GO:0030017> | 1 | Cellular component | sarcomere |
| <GO:0030018> | 1 | Cellular component | Z disc |
| <GO:0031674> | 1 | Cellular component | I band |
| <GO:0032153> | 1 | Cellular component | cell division site |
| <GO:0033270> | 1 | Cellular component | paranode region of axon |
| <GO:0034399> | 1 | Cellular component | nuclear periphery |
| <GO:0034451> | 1 | Cellular component | centriolar satellite |
| <GO:0043203> | 1 | Cellular component | axon hillock |
| <GO:0044304> | 1 | Cellular component | main axon |
| <GO:0048786> | 1 | Cellular component | presynaptic active zone |
| <GO:0051233> | 1 | Cellular component | spindle midzone |
| <GO:0061827> | 1 | Cellular component | sperm head |
| <GO:0070971> | 1 | Cellular component | endoplasmic reticulum exit site |
| <GO:0090543> | 1 | Cellular component | Flemming body |
| <GO:0090734> | 1 | Cellular component | site of DNA damage |
| <GO:0097225> | 1 | Cellular component | sperm midpiece |
| <GO:0097542> | 1 | Cellular component | ciliary tip |
| <GO:1990876> | 1 | Cellular component | cytoplasmic side of nuclear pore |
| <GO:0005515> | 126 | Molecular function | protein binding |
| <GO:0043167> | 102 | Molecular function | ion binding |
| <GO:0097159> | 93 | Molecular function | organic cyclic compound binding |
| <GO:1901363> | 93 | Molecular function | heterocyclic compound binding |
| <GO:0016787> | 65 | Molecular function | hydrolase activity |
| <GO:0140096> | 57 | Molecular function | catalytic activity, acting on a protein |
| <GO:0036094> | 52 | Molecular function | small molecule binding |
| <GO:0097367> | 47 | Molecular function | carbohydrate derivative binding |
| <GO:0016740> | 45 | Molecular function | transferase activity |
| <GO:0022857> | 20 | Molecular function | transmembrane transporter activity |
| <GO:0038023> | 19 | Molecular function | signaling receptor activity |
| <GO:0003700> | 16 | Molecular function | DNA-binding transcription factor activity |
| <GO:0044877> | 14 | Molecular function | protein-containing complex binding |
| <GO:0016491> | 13 | Molecular function | oxidoreductase activity |
| <GO:0030234> | 12 | Molecular function | enzyme regulator activity |
| <GO:0140677> | 12 | Molecular function | molecular function activator activity |
| <GO:0140678> | 11 | Molecular function | molecular function inhibitor activity |
| <GO:0005200> | 10 | Molecular function | structural constituent of cytoskeleton |
| <GO:0008289> | 9 | Molecular function | lipid binding |
| <GO:0140640> | 6 | Molecular function | catalytic activity, acting on a nucleic acid |
| <GO:0003682> | 6 | Molecular function | chromatin binding |
| <GO:0016887> | 6 | Molecular function | ATP hydrolysis activity |
| <GO:0003712> | 5 | Molecular function | transcription coregulator activity |
| <GO:0016829> | 4 | Molecular function | lyase activity |
| <GO:0003735> | 4 | Molecular function | structural constituent of ribosome |
| <GO:0016247> | 4 | Molecular function | channel regulator activity |
| <GO:0016874> | 3 | Molecular function | ligase activity |
| <GO:0033218> | 3 | Molecular function | amide binding |
| <GO:0008094> | 3 | Molecular function | ATP-dependent activity, acting on DNA |
| <GO:0003777> | 2 | Molecular function | microtubule motor activity |
| <GO:0016853> | 2 | Molecular function | isomerase activity |
| <GO:0005319> | 2 | Molecular function | lipid transporter activity |
| <GO:0030246> | 2 | Molecular function | carbohydrate binding |
| <GO:0042562> | 2 | Molecular function | hormone binding |
| <GO:0050997> | 2 | Molecular function | quaternary ammonium group binding |
| <GO:1901681> | 2 | Molecular function | sulfur compound binding |
| <GO:0030545> | 2 | Molecular function | signaling receptor regulator activity |
| <GO:0004386> | 2 | Molecular function | helicase activity |
| <GO:0042626> | 2 | Molecular function | ATPase-coupled transmembrane transporter activity |
| <GO:0017056> | 1 | Molecular function | structural constituent of nuclear pore |
| <GO:0030527> | 1 | Molecular function | structural constituent of chromatin |
| <GO:0048038> | 1 | Molecular function | quinone binding |
| <GO:0051540> | 1 | Molecular function | metal cluster binding |
| <GO:0004694> | 1 | Molecular function | eukaryotic translation initiation factor 2alpha kinase activity |
| <GO:0090079> | 1 | Molecular function | translation regulator activity, nucleic acid binding |
| <GO:0030674> | 1 | Molecular function | protein-macromolecule adaptor activity |
| <GO:0140142> | 1 | Molecular function | nucleocytoplasmic carrier activity |
| <GO:0140612> | 1 | Molecular function | DNA damage sensor activity |
| <GO:0036402> | 1 | Molecular function | proteasome-activating activity |
| <GO:0140326> | 1 | Molecular function | ATPase-coupled intramembrane lipid transporter activity |

**Table S6:** Results of a linear mixed model comparing the wealth of the first breeding territory of all returning adults born in our population to the wealth of their natal territory, in relationship to dispersal distance (i.e. the distance between their natal and first breeding territories).

|  | **Breeding wealth of all recruiting individuals** | | | |  |  |
| --- | --- | --- | --- | --- | --- | --- |
| *fixed effects* | *estimate* | *std.error* | *t-value* | *p-value* |  |  |
| Intercept | 0.00 | 0.03 | 0.11 | 0.910 |  |  |
| natal wealth | 0.25 | 0.03 | 8.18 | **<0.001** |  |  |
| dispersal distance | 0.04 | 0.04 | 1.25 | 0.212 |  |  |
| natal wealth x dispersal distance | -0.11 | 0.03 | -3.79 | **<0.001** |  |  |
| **Random Effects** *Variance N* | | | | |  |  |
| Residuals | 0.93 | | | |  |  |
| Natal nest | 0.00 776 | | | |  |  |

**References cited in this supplementary document**

Arnold, K. E., Ramsay, S. L., Henderson, L., & Larcombe, S. D. (2010). Seasonal variation in diet quality: antioxidants, invertebrates and blue tits Cyanistes caeruleus. *Biological Journal of the Linnean Society*, *99*(4), 708–717. https://doi.org/10.1111/J.1095-8312.2010.01377.X

Aulchenko, Y. S., Ripke, S., Isaacs, A., & van Duijn, C. M. (2007). GenABEL: an R library for genome-wide association analysis. *Bioinformatics*, *23*(10), 1294–1296. https://doi.org/10.1093/BIOINFORMATICS/BTM108

Bitterlich, W. (1984). *The relascope idea: relative measurements in forestry*. Commonwealth Agricultural Bureaux.

Burger, C., Belskii, E., Eeva, T., Laaksonen, T., Mägi, M., Mänd, R., Qvarnström, A., Slagsvold, T., Veen, T., Visser, M. E., others, Wiebe, K. L., Wiley, C., Wright, J., & Both, C. (2012). Climate change, breeding date and nestling diet: how temperature differentially affects seasonal changes in pied flycatcher diet depending on habitat variation. *Journal of Animal Ecology*, *81*(4), 926–936. https://doi.org/10.1111/j.1365-2656.2012.01968.x

Cimmery, V. (2007). *SAGA User Guide, Updated for SAGA Version 2.0.5.*

Doligez, B., Danchin, E., & Clobert, J. (2002). Public information and breeding habitat selection in a wild bird population. *Science*, *297*(5584), 1168–1170. https://doi.org/10.1126/SCIENCE.1072838/SUPPL_FILE/DOLIGEZSUPPL.PDF

Geweke, J. (1991). Evaluating the accuracy of sampling-based approaches to the calculation of posterior moments. In *Staff Report*. Federal Reserve Bank of Minneapolis. https://ideas.repec.org/p/fip/fedmsr/148.html

Hadfield, J. D. (2010). MCMC Methods for Multi-Response Generalized Linear Mixed Models: The MCMCglmm R Package. *Journal of Statistical Software*, *33*(2), 1–22. https://doi.org/10.18637/JSS.V033.I02

Hastie, T., Tibshirani, R., & Friedman, J. (2009). *The Elements of Statistical Learning Data Mining, Inference, and Prediction* (2nd ed.). Springer. https://doi.org/https://doi.org/10.1007/978-0-387-84858-7

Heidelberger, P., & Welch, P. D. (1983). Simulation Run Length Control in the Presence of an Initial Transient. *Operations Research*, *31*(6), 1109–1144. https://doi.org/10.1287/OPRE.31.6.1109

Huhta, E., Jokimakp, J., & Rahko, P. (1998). Distribution and reproductive success of the Pied Flycatcher Ficedula hypoleuca in relation to forest patch size and vegetation characteristics; the effect of scale. *Ibis*, *140*(2), 214–222. https://doi.org/10.1111/j.1474-919X.1998.tb04382.x

Linden, M., Gustafsson, L., & Part, T. (1992). Selection on Fledging Mass in the Collared Flycatcher and the Great Tit. *Ecology*, *73*(1), 336–343. https://doi.org/10.2307/1938745

Nadolski, J., Marciniak, B., Loga, B., Michalski, M., & Bańbura, J. (2021). Long-term variation in the timing and height of annual peak abundance of caterpillars in tree canopies: Some effects on a breeding songbird. *Ecological Indicators*, *121*, 107120. https://doi.org/10.1016/J.ECOLIND.2020.107120

Olaya, V. (2004). *A Gentle Introduction to SAGA GIS* (V. Olaya, Ed.).

Pärt, T. (1991). Philopatry Pays: A Comparison between Collared Flycatcher Sisters. *The American Naturalist*, *138*(3), 790–796. https://doi.org/10.1086/285252

Pärt, T. (1994). Male philopatry confers a mating advantage in the migratory collared flycatcher, Ficedula albicollis. *Animal Behaviour*, *48*(2), 401–409. https://doi.org/10.1006/ANBE.1994.1254

Pärt, T., & Qvarnström, A. (1997). Badge size in collared flycatchers predicts outcome of male competition over territories. *Animal Behaviour*, *54*(4), 893–899. https://doi.org/10.1006/anbe.1997.0514

Pommerening, A., & Sterba, H. (2024). Monitoring spatial tree diversity indices using relascope sampling: Estimators, interactions and limitations. *Ecological Informatics*, *81*, 102579. https://doi.org/10.1016/J.ECOINF.2024.102579

Purcell, S., Neale, B., Todd-Brown, K., Thomas, L., Ferreira, M. A. R., Bender, D., Maller, J., Sklar, P., De Bakker, P. I. W., Daly, M. J., & Sham, P. C. (2007). PLINK: a tool set for whole-genome association and population-based linkage analyses. *American Journal of Human Genetics*, *81*(3), 559–575. https://doi.org/10.1086/519795

Rönnegård, L., McFarlane, S. E., Husby, A., Kawakami, T., Ellegren, H., & Qvarnström, A. (2016). Increasing the power of genome wide association studies in natural populations using repeated measures – evaluation and implementation. *Methods in Ecology and Evolution*, *7*(7), 792–799. https://doi.org/10.1111/2041-210X.12535

Rybinski, J., Sirkiä, P. M., McFarlane, S. E., Vallin, N., Wheatcroft, D., Ålund, M., & Qvarnström, A. (2016). Competition-driven build-up of habitat isolation and selection favoring modified dispersal patterns in a young avian hybrid zone. *Evolution*, *70*(10), 2226–2238. https://doi.org/10.1111/evo.13019

Siikamäki Pirkko. (1998). Limitation of reproductive success by food availability and breeding time in pied flycatchers. *Ecology*, *79*(5), 1789–1796. https://doi.org/10.1890/0012-9658(1998)079[1789:LORSBF]2.0.CO;2

Silva, C. N. S., McFarlane, S. E., Hagen, I. J., Rönnegård, L., Billing, A. M., Kvalnes, T., Kemppainen, P., Rønning, B., Ringsby, T. H., Sæther, B. E., Qvarnström, A., Ellegren, H., Jensen, H., & Husby, A. (2017). Insights into the genetic architecture of morphological traits in two passerine bird species. *Heredity 2017 119:3*, *119*(3), 197–205. https://doi.org/10.1038/hdy.2017.29

Simon, N., Friedman, J., Hastie, T., & Tibshirani, R. (2011). Regularization paths for Cox’s proportional hazards model via coordinate descent. *Journal of Statistical Software*, *39*(5), 1–13. https://doi.org/10.18637/JSS.V039.I05

Sirkiä, P. M., McFarlane, S. E., Jones, W., Wheatcroft, D., Ålund, M., Rybinski, J., & Qvarnström, A. (2018). Climate-driven build-up of temporal isolation within a recently formed avian hybrid zone. *Evolution*, *72*(2), 363–374. https://doi.org/10.1111/evo.13404

Slagsvold, T., & Wiebe, K. L. (2007). Hatching asynchrony and early nestling mortality: the feeding constraint hypothesis. *Animal Behaviour*, *73*(4), 691–700. https://doi.org/10.1016/J.ANBEHAV.2006.05.021

Van Asch, M., & Visser, M. E. (2007). Phenology of forest caterpillars and their host trees: The importance of synchrony. *Annual Review of Entomology*, *52*(Volume 52, 2007), 37–55. https://doi.org/10.1146/ANNUREV.ENTO.52.110405.091418/CITE/REFWORKS

Van Balen, J. H. (1973). A Comparative Sudy of the Breeding Ecology of the Great Tit Parus major in Different Habitats. *Ardea*, *55*(1–2), 1–93. https://doi.org/10.5253/ARDE.V61.P1

Veen, T., Sheldon, B. C., Weissing, F. J., Visser, M. E., Qvarnström, A., & Sætre, G.-P. (2010). Temporal differences in food abundance promote coexistence between two congeneric passerines. *Oecologia*, *162*(4), 873–884.

Visser, M. E., Holleman, L. J. M., & Gienapp, P. (2006). Shifts in caterpillar biomass phenology due to climate change and its impact on the breeding biology of an insectivorous bird. *Oecologia*, *147*(1). https://doi.org/10.1007/s00442-005-0299-6
